# Tumor Microenvironment Cascade-Responsive Nanodrug with Self-Targeting Activation and ROS Regeneration for Synergistic Oxidation-Chemotherapy

**DOI:** 10.1007/s40820-020-00492-4

**Published:** 2020-09-14

**Authors:** Yang Li, Jinyan Lin, Peiyuan Wang, Qiang Luo, Fukai Zhu, Yun Zhang, Zhenqing Hou, Xiaolong Liu, Jingfeng Liu

**Affiliations:** 1 CAS Key Laboratory of Design and Assembly of Functional Nanostructures, Fujian Institute of Research on the Structure of Matter, Chinese Academy of Sciences, Fuzhou, 350002 People’s Republic of China; 2 The United Innovation of Mengchao Hepatobiliary Technology Key Laboratory of Fujian Province, Mengchao Hepatobiliary Hospital of Fujian Medical University, Fuzhou, 350025 People’s Republic of China; 3 Department of Translational Medicine, Xiamen Institute of Rare Earth Materials, Chinese Academy of Sciences, Xiamen, 361024 People’s Republic of China; 4 College of Materials, Xiamen University, Xiamen, 361005 People’s Republic of China

**Keywords:** Targeting activation, Positive-feedback loop, Circular amplification of ROS, Vitamin E nanodrug, Synergistic oxidation-chemotherapy

## Abstract

Carrier-free nanodrug with exceptionally high drug payload has attracted increasing attentions. Herein, we construct a pH/ROS cascade-responsive nanodrug which could achieve tumor acidity-triggered targeting activation followed by circularly amplified ROS-triggered drug release via positive-feedback loop. The di-selenide-bridged prodrug synthesized from vitamin E succinate and methotrexate (MTX) self-assembles into nanoparticles (VSeM); decorating acidity-cleavable PEG onto VSeM surface temporarily shields the targeting ability of MTX to evade immune clearance and consequently elongate circulation time. Upon reaching tumor sites, acidity-triggered detachment of PEG results in targeting recovery to enhance tumor cell uptake. Afterward, the VSeM could be dissociated in response to intracellular ROS to trigger VES/MTX release; then the released VES could produce extra ROS to accelerate the collapse of VSeM. Finally, the excessive ROS produced from VES could synergize with the released MTX to efficiently suppress tumor growth via orchestrated oxidation-chemotherapy. Our study provides a novel strategy to engineer cascade-responsive nanodrug for synergistic cancer treatment.

**Electronic supplementary material:**

The online version of this article (10.1007/s40820-020-00492-4) contains supplementary material, which is available to authorized users.

## Highlights

- A pH/ROS cascade-responsive carrier-free nanodrug with self-targeting activation and ROS regeneration abilities.
- On-demand release of VES in response to intracellular ROS to accelerate drug release via positive-feedback loop.
- Highly synergistic therapeutic efficiency via orchestrated cooperation of oxidation-chemotherapy.

**Electronic supplementary material:**

The online version of this article (10.1007/s40820-020-00492-4) contains supplementary material, which is available to authorized users.

## Graphical abstract

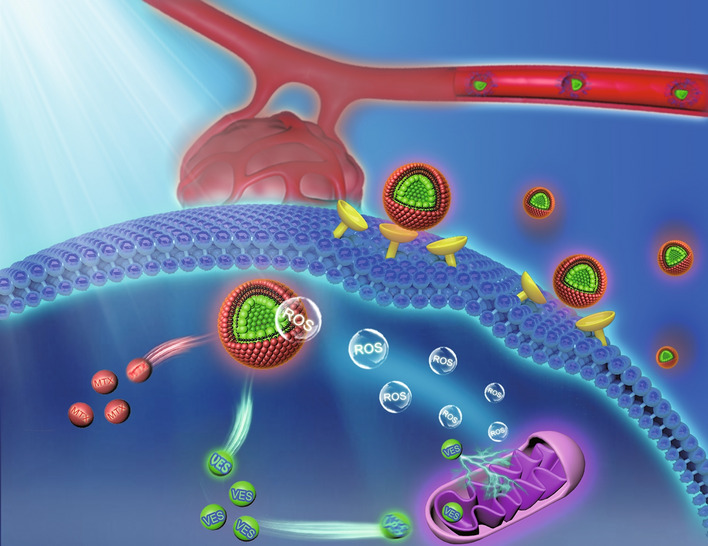

## Introduction

Integrating advantages of carrier-free nanodrug (e.g., exceptionally high drug payload and avoidance of possible toxicity and immunogenicity caused by carrier materials) [1, 2] and prodrug (e.g., controlled release) [3, 4], carrier-free nano-prodrug has emerged as a promising alternative strategy to circumvent the obstacles of traditional chemotherapy (e.g., poor tumor selectivity and toxic side effects) and improve the anticancer effects [5–7]. Taking the advantage of enhanced permeability and retention (EPR) effect, carrier-free nano-prodrug could remarkably improve the transport efficiency of anticancer drug to tumor sites, hence enhancing the bioavailability and therapeutic efficacy [8, 9]. Insufficient accumulation of carrier-free nano-prodrug both at tumor sites and within tumor cells remains one of the significant challenges for preclinical translation [10, 11]. Meanwhile, inadequate controllability for drug release within tumors over surrounding normal tissues further limits the therapeutic efficacy while increasing the toxicity [12–14]. To improve therapeutic efficacy and minimize undesirable toxicity, considerable efforts have been devoted to develop tumor-targeting stimuli-triggered nano-systems [15–18]. On one hand, the nano-systems should keep their stealth function in blood circulation but sequentially undergo a transformation process once reaching at tumor sites for enhancing the binding to tumor cells to increase cellular uptake [10, 11]. Owing to the weakly acidic feature of tumor microenvironment, tumor acidity-triggered active targeting activation strategy based on pH-responsive dePEGylation and re-exposure of targeting ligand was typically utilized [11], which could temporarily shield targeting function under physiological environment (pH 7.4) to evade the immune clearance and improve the circulation time, while the targeting function could be recovered once exposing to weakly acidic tumor microenvironment (pH 6.5–6.8) to enhance tumor cell uptake [19, 20]. However, almost all foreign ligands rarely possess any therapeutic function by themselves [21]. It could be imagined that developing a self-targeting carrier-free nano-prodrug without foreign ligand would be rather more attractive to satisfy both targeting and therapeutic needs while avoiding additional design complexity. On the other hand, after internalization into cells, the nano-systems should display on-demand drug release by responding to the internal or external stimuli [22, 23]. In contrast to lysosomal acidic pH and intracellular high glutathione level which exist both in normal and cancer cells, the level of reactive oxygen species (ROS) involving hydrogen peroxides (H_2_O_2_), superoxide anion (·O_2_^−^), and hydroxyl radicals (·OH) in cancer cells (50–100 × 10^−6^ M) is far more higher compared to that in normal cells (~ 20 × 10^−9^ M) [24, 25]; thus, nano-system with ROS-responsive characteristic is a promising alternative to specifically release drug within tumor cells. Diversified ROS-cleavable group including thioether, thioketal, phenylboronic ester, peroxalate ester, and selenium/tellurium have been widely utilized to integrate within drug delivery systems [26, 27]. However, the endogenous ROS level is still not high enough to sufficiently trigger the complete decomposition, therefore not enough for adequate drug release (required ROS level window: 1–100 × 10^−3^ M) [28, 29]. Furthermore, the endogenous ROS level is also hugely varied among cancer cells due to the tumor cell heterogeneity [30]. Therefore, it would be very important to in situ amplify the ROS levels to accelerate drug release and overcome tumor heterogeneity on-demand.

**Scheme 1:**
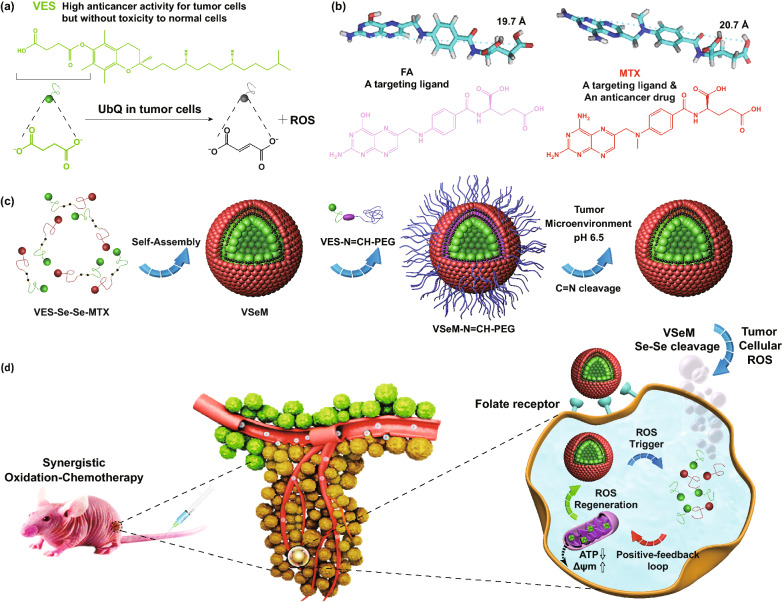
**a** Chemical structure of VES and ROS production mechanism of VES by specifically interacting with ubiquinone (UbQ)-binding site in complex II of the mitochondrial electron transport chain in tumor cells. **b** Optimized molecular structures and chemical structures of FA and MTX (FA analogue). MTX is structurally similarity to FA regardless a key feature that MTX possesses an amino group whereas FA possesses a hydroxyl group at the 4-position of pteridine ring. **c** Synthesis routes of ROS-responsive VES-Se-Se-MTX and tumor acidity-responsive VES-N=CH-PEG. **d** Illustration of pH/ROS cascade-responsive VSeM-N=CH-PEG nano-prodrug with tumor acidity-triggered active self-targeting recovery and amplified ROS-triggered drug release for synergistic oxidation-chemotherapy

Vitamin E succinate (VES, a succinyl derivative of vitamin E, Scheme 1a) has be recognized as a carrier skeleton for loading drugs, and at the meantime it also could be utilized as a therapeutic molecule due to its intrinsic anticancer activity [31–33], which has been proved in preliminary clinical trials [34–36]. Interestingly, the VES could further interact with the ubiquinone (UbQ)-binding site in mitochondrial respiratory chain Complex II to interfere the electron transport chain for rapid and preferential ROS generation [31–33], and this process accompanied with the consumption of adenosine triphosphate (ATP) which could increase the chemotherapeutic sensitization of tumor cells to synergistically augment anti-tumor efficiency of chemotherapeutic drugs [37]. Thus, we inferred that introducing VES in ROS-triggered nano-prodrug can selectively and circularly amplify intracellular ROS in tumors to realize rapid and adequate drug release as well as synergistic therapy.

In view of that bi-functional targeting and anticancer candidate molecules attracted increasing attention for developing a simplified yet multifunctional nano-prodrug, we were greatly motivated and interested in methotrexate (MTX, Scheme 1b), which not only possess anti-tumor function but also show innate affinity toward folate receptors [38, 39]. Fortunately, our preliminary experiments demonstrated that both VES and MTX could exert a synergy in anti-tumor effectiveness against HeLa and MCF-7 cells via cytotoxicity assessment and combination index (CI) analysis using a classic isobologram equation of Chou-Talalay (Fig. S1).

Herein, we constructed a pH/ROS cascade-responsive vitamin E nanodrug which could achieve tumor acidity-triggered self-targeting activation followed by circularly amplified ROS-triggered drug release via positive-feedback loop (Scheme 1c, d). Firstly, the VES and the MTX were selected to synthesize VES-Se-Se-MTX prodrug via di-selenium linkage, which could self-assemble into VSeM nano-prodrug in aqueous solution by di-selenium-induced small-molecule assembly [40, 41]. Sequentially, the surface of VSeM nano-prodrug was functionalized with tumor acidity-responsive VES-N=CH-PEG which was synthesized by dynamic covalent benzoic-imine linkage. Theoretically, this nano-prodrug could provide practical benefits in a programmable manner: (1) the PEG shell could temporarily shield targeting function, and thus evade the immune clearance and prolong the circulation time; (2) once exposed to tumor microenvironment, the intrinsic weak acidity could trigger the detachment of PEG shell to re-expose the MTX ligand on the surface, which could lead to the recovery of self-targeting to recognize folate receptor and therefore promote the tumor cell internalization; (3) after uptake into tumor cells, the inherent ROS only could partially trigger disassembly of VSeM core to release a certain amount of VES by cleaving the di-selenium linkage; (4) the released VES could interfere the electron transport chain to produce additional ROS and consume ATP, thus resulting in the acceleration of core collapse and drug release via positive-feedback loop. Finally, the VES could synergize with MTX to aggravate tumor cell killing. Thus, our designed tumor microenvironment-activated self-recognizing and ROS-generating vitamin E nanodrug might provide a promising candidate for synergistically improving oxidation-chemotherapeutic efficiency.

## Experimental and Characterization

### Materials

Vitamin E succinate (VES), methotrexate (MTX), folic acid (FA), 4-carboxybenzaldehyde (*p*-CBA), N-hydroxysuccinimide (NHS), 1-hydroxybenzotriazole (HOBt), dimethylaminopyridine (DMAP), and 1-(3-dimethylaminopropyl)-3-ethylcarbodiimide hydrochloride (EDC·HCl) were obtained from Aladdin Chemistry Co., Ltd. (China). MEPG-OH (*M*_*w*_ ~ 2000 Da) was purchased from XiaMen Sinopeg Biotech Co., Ltd. (China). Selenocystamine dihydrochloride (Se-CYS·HCl) was purchased from Accelerating Scientific and Industrial Development thereby Serving Humanity (China). 3-(4, 5-dimethyl-thiazol-2-yl)-2, 5-diphenyltetrazolium bromide (MTT) was purchased from Sigma-Aldrich (USA). 2′, 7′-dichlorofluorescein diacetate (DCFH-DA), LysoTracker Green DND-26, and Hoechst 33258 were supplied by Beyotime Biotechnology Co., Ltd. (China). Fetal bovine serum (FBS), penicillin–streptomycin, Dulbecco’s modified Eagle’s medium (DMEM), and trypsin were purchased from Gibco Life Technologies (AG, Switzerland). All chemical reagents of analytical grade and used as received.

### Characterization

Transmission electron microscopy (TEM) imaging was performed on a JEOL JEM-1400 electron microscope (Japan). Scanning electron microscopy (SEM) imaging was performed on a LEO1530VP SEM (Germany). UV–Vis-NIR absorption spectra were acquired from a UV-3600 plus UV–Vis-NIR spectrometer (Japan). Fourier transform infrared spectroscopy (FT-IR) spectra were collected on a Bruker IFS-55 infrared spectrometer (Bruker, Switzerland). Fluorescence spectra were obtained from a FluoroMax-4 spectrophotometer (USA). Nuclear magnetic resonance (NMR) spectra were recorded on a Bruker AV400 MHz NMR spectrometer (USA). High-resolution mass spectra (MS) were recorded with a Bruker Apex Ultra 7.0 FT-MS mass spectrometer. Matrix-assisted laser desorption/ionization time-of-flight mass (MALDI-TOF MS) was conducted on a Bruker Microflex LRF mass spectrometer. Dynamic light scattering (DLS) and zeta potential measurements were carried out on a Malvern Zetasizer. Confocal laser scanning microscopy (CLSM) imaging was performed with a Leica TCS SP5 confocal laser scanning microscopy (Germany). Fluorescence imaging was performed by an IVIS Lumina imaging system (USA). Photoacoustic imaging was carried out using a VisualSonics Vevo-2100 system (Canada).

### Synthesis of ROS-Responsive VES-Se-Se-MTX

VES-Se-Se-MTX was synthesized between VES and MTX via the bridge of Se-CYS with EDC/NHS catalysis. For the synthesis of VES-CYS, VES (53.0 mg), EDC·Cl (57.4 mg), and NHS (34.5 mg) were dissolved in 10 mL of dimethylsulfoxide and stirred for 4 h to activate the carboxyl of VES. Afterward, Se-CYS·Cl (47.8 mg) dissolved in dimethylsulfoxide/deionized water (20: 1, v/v) was added dropwise to the above solution and stirred for 24 h under an argon atmosphere. The resultant compound was dialyzed with deionized water (MWCO = 3500 Da), centrifugated, washed, and lyophilized. For the synthesis of VES-CYS-MTX, MTX (68.1 mg), EDC·Cl (86.1 mg), and NHS (51.7 mg) were dissolved in 10 mL of dimethylsulfoxide and stirred for 4 h to activate the carboxyl of MTX. Subsequently, the synthesized VES-CYS dissolved in dimethylsulfoxide was added dropwise to the above solution and stirred for 24 h under an argon atmosphere. The resultant compound was dialyzed with deionized water (MWCO = 3500 Da) and lyophilized. The productivity was estimated to be ~ 64%.

### Synthesis of pH-Responsive VES-N=CH-PEG

MPEG-CHO was synthesized between MPEG-OH and *p*-CBA via EDC/DMAP catalysis. VES-NH_2_ was also synthesized between VES and ethanediamine via EDC/HOBt catalysis. Afterward, VES-N=CH-PEG was synthesized between MPEG-CHO and VES-NH_2_ via benzoic-imine linker. In brief, VES-NH_2_ (85.8 mg) and MPEG-CHO (213.2 mg) were dissolved in 20 mL of dichloromethane and stirred for 24 h under an argon atmosphere. Afterward, the reaction mixture was rotary-evaporated to remove dichloromethane. After adding ice-cold diethyl ether/acetone, the resultant was precipitated to separate from unreacted and excess VES-NH_2_. Lastly, the resultant VES-N=CH-PEG was re-dispersed in ethyl alcohol/deionized water (1: 1, v/v) for dialyzing with deionized water (MWCO = 3500 Da) followed by lyophilization.

### Preparation of VSeM-N=CH-PEG

The VSeM-N=CH-PEG was prepared by self-assembly technique followed by surface insertion method. Briefly, 10 mg of VES-Se-Se-MTX was dissolved in 2 mL of dimethylsulfoxide, and 10 mL of deionized water was added dropwise. After ultrasonication at 800 W for 10 min and stirring for 2 h at room temperature, the VSeM nanodrugs were obtained by di-selenium bridge-induced assembly. Then, 2 mg of VES-N=CH-PEG was added to the obtained VSeM dispersions. After ultrasonication at 200 W for 10 min and stirring for 6 h, the resultant VSeM-N=CH-PEG nanodrugs was centrifugated, washed, and re-dispersed in deionized water. Besides, the VSeM-PEG nanodrugs were prepared with the similar method except that pH-responsive VES-N=CH-PEG should be replaced by pH-unresponsive VES-PEG. For fluorescence labeling, the DiD/DiR-labeled VSeM-N=CH-PEG nanodrugs were prepared with the similar method except that 10 mg of VES-Se-Se-MTX should be replaced by 10 mg of VES-Se-Se-MTX and 0.2 mg of DiD/DiR.

### In Vitro Drug Release

Typically, 2 mL of ROS-unresponsive VCM-N=CH-PEG or ROS-responsive VSeM-N=CH-PEG (1 mg mL^−1^) were transferred into a dialysis bag (MWCO = 3, 500), and then immersed within 60 mL of PBS without or with H_2_O_2_ (0, 0.1, 1, and 10 mM) at 37 °C in a beaker flask. At predetermined time intervals, 2.0 mL of external PBS was collected and then replaced with 2.0 mL of fresh PBS. Finally, the concentration of MTX was determined by HPLC analysis.

### In Vitro Cellular Uptake

The cellular uptake was studied in HeLa and MCF-7 cells by using CLSM and flow cytometry. For CLSM observation, HeLa cells were seeded in 6-well plates at 1.0 × 10^5^ cells per well and cultured for 24 h. Subsequently, the DiD-labeled VSeM-PEG or VSeM-N=CH-PEG was added to different wells and the cells were incubated at pH 7.4 or 6.5 for different times. After incubation, the cells were orderly washed thrice with PBS, fixed with 4% formaldehyde for 20 min, stained with Hoechst33258 for 10 min, and visualized by a Leica TCS SP5 CLSM. For flow cytometry analysis, HeLa cells were seeded in 6-well plates at 5.0 × 10^5^ cells per well and cultured for 24 h. Subsequently, the DiD-labeled VSeM-PEG or VSeM-N=CH-PEG was added to different wells and the cells were incubated for different time periods. Finally, the cells were washed thrice with PBS, harvested with trypsin, and analyzed by a FACSCalibur flow cytometer.

### In Vitro ROS Detection

HeLa cells were seeded in 6-well plates at 1.0 × 10^5^ cells per well and cultured for 24 h. Subsequently, the cells were incubated with VES, VES/MTX, VCM-N=CH-PEG, VSeM-PEG, and VSeM-N=CH-PEG at pH 6.5 for 4 h and then washed thrice with PBS. At the end of incubation, the DCFH-DA fluorescent dye was added and co-incubated for 20 min. Finally, the cells were washed thrice and imaged by a Leica TCS SP5 CLSM with 488 nm excitation. Besides, the cells were washed thrice with PBS, harvested with trypsin, and assayed using a FACSCalibur flow cytometer.

### In Vitro Mitochondrial Membrane Potential Detection

HeLa cells were seeded in 6-well plates at 1.0 × 10^5^ cells per well and cultured for 24 h. Subsequently, the cells were incubated with VES/MTX, VCM-N=CH-PEG, VSeM-PEG, and VSeM-N=CH-PEG at pH 6.5 for 12 h. At the end of incubation, the cells were washed thrice with PBS. Subsequently, the JC-1 fluorescent probe was added and co-incubated for 30 min. Finally, the cells were washed thrice and the mitochondrial damage/disruption was detected by a Leica TCS SP5 CLSM and a FACSCalibur flow cytometer.

### In Vitro Cytotoxicity

The cell *via*bility was evaluated with MTT assay. HeLa and MCF-7 cells were seeded into 96-well plates at 1 × 10^4^ cells per well and cultured for 24 h. Subsequently, the medium was removed and replaced with 200 μL of complete medium containing different concentrations of VES/MTX, VCM-N=CH-PEG, VSeM-PEG, and VSeM-N=CH-PEG at pH 6.5 in the presence of 10 μM H_2_O_2_. On the other hand, HeLa and MCF-7 cells were incubated with different concentrations of VSeM-PEG and VSeM-N=CH-PEG at pH 7.4 or 6.5 for tumor acidity-triggered cytotoxicity assay. After 24 h of incubation, the cells were washed thrice with PBS, and 150 μL of MTT solution (0.5 mg mL^−1^) was then added to each well. After 4 h of treatment, the MTT solution was discarded, and 150 μL of dimethylsulfoxide was added to dissolve crystals. Finally, the absorbance was recorded at 490 nm by a microplate reader.

### In Vitro Apoptosis

HeLa cells were seeded in 6-well plates at 2.0 × 10^5^ cells per well and cultured for 24 h. Subsequently, the cells were incubated with VES/MTX, VCM-N=CH-PEG, VSeM-PEG, and VSeM-N=CH-PEG at pH 6.5 for 12 h. At the end of incubation, the cells were harvested, washed thrice with ice-cold PBS, and stained with Annexin V and PI using an Annexin V-FITC/PI apoptosis detection Kit according to the manufacturer’s instruction. Finally, the cell apoptosis was analyzed by a FACSCalibur flow cytometer.

### In Vitro Live/Dead Cell Staining

HeLa cells were seeded in 12-well plates at 1.0 × 10^5^ cells per well and cultured for 12 h. Subsequently, the cells were incubated with VES/MTX, VCM-N=CH-PEG, VSeM-PEG, and VSeM-N=CH-PEG at pH 6.5 for 12 h. At the end of incubation, the culture medium was discarded and the cells were washed thrice with PBS. Afterward, the cells were stained with calcein-AM and PI using a calcein-AM/PI staining Kit for 20 min. Finally, the cells were washed thrice with PBS and imaged by CLSM. Green fluorescence of calcein-AM was excited at 488 nm and detected with a 500–550 nm bandpass filter. Red fluorescence of PI was excited at 633 nm and detected with a 660–710 nm bandpass filter.

### Animals and Tumor Models

All procedures of animal study were approved by the Institutional Animal Care and Use Committee of Xiamen University. BALB/c female nude mice (4–6 weeks old, ~ 18 g) were supplied by Experimental Animal Laboratory of Cancer Research Center of Xiamen University. 100 µL of cell suspensions containing 5 × 10^6^ HeLa cells were injected subcutaneously in the right flank region of BALB/c female nude mice. The tumors were allowed to reach approximately 100–150 mm^3^ before subsequent experiments.

### In Vivo Pharmacokinetics

The HeLa tumor-bearing BALB/c mice were intravenously injected with 200 μL of VES/MTX, VSeM, VSeM-PEG, and VSeM-N=CH-PEG at the same concentration of MTX (4 mg kg^−1^), respectively. The tail blood was taken at determined time points (30 min, 1, 2, 4, 8, 10, 12, and 24 h) and centrifuged at 3000 rpm for 15 min. Afterward, the obtained plasma was treated with acetonitrile/methanol mixture (1: 1, v/v) and separated by centrifugation for determination of MTX concentration by using high-performance liquid chromatography (HPLC) method.

### In Vivo Biodistribution

The HeLa tumor-bearing BALB/c mice were intravenously injected with 200 µL of VES/MTX, VSeM, VSeM-PEG, and VSeM-N=CH-PEG at the same concentration of MTX (4 mg kg^−1^), respectively. At different time points, the mice were sacrificed to excise various tissues including the heart, liver, spleen, lung, kidney, and tumor. Subsequently, the tissues were homogenized with acetonitrile/methanol mixture (1: 1, v/v) and separated by centrifugation for determination of MTX concentration using high-performance liquid chromatography (HPLC) method.

### In Vivo Fluorescence Imaging

The HeLa tumor-bearing nude mice were injected with 200 μL of free DiR, DiR-labeled VSeM, VSeM-PEG, VSeM-N=CH-PEG, and VSeM-N=CH-PEG + free FA at the same concentration of DiR, respectively, via tail vein. The fluorescence imaging was acquired an IVIS Lumina imaging system at different time points (1, 2, 4, 8, 24, and 48 h). At 48 h post-injection of Cy5.5-labeled VSeM-N=CH-PEG, the mice were sacrificed, and the organs and tumor tissue were excised for ex vivo fluorescence imaging and semi-quantitative analysis.

### In Vivo Anti-tumor Effect

The HeLa tumor-bearing mice were divided to five groups (*n* = 6 per group): (I) PBS, (II) VES/MTX, (III) VCM-PEG, (IV) VCM-N=CH-PEG, (V) VSeM-PEG, and (VI) VSeM-N=CH-PEG. Each mouse of different group was earmarked and followed individually throughout the whole experiments. The tumor sizes and body weights were measured every day until the test was ended. Tumor volume (*V*) was calculated using the formula: *V*(mm^3^) = 1/2 × length (mm) × width (mm)^2^. After 14 days, the mice were sacrificed. Subsequently, the tumors were separated, weighted, and photographed. For histopathologic analysis, the separated tumors and major organs (heart, liver, spleen, lung, and kidney) were fixed in 10% formalin, embedded in paraffin, and sectioned at 8 mm for hematoxylin–eosin (H&E), TUNEL, and Ki-67 staining assay.

### Statistical Analysis

Statistical evaluations of data were performed using the one-way analysis of variance (ANOVA). All results were expressed as mean ± standard error unless otherwise noted, in which *P *< 0.05 (*) was significant, *P *< 0.01 (**) was very significant, and *P *< 0.001 (***) was highly significant.

## Results and Discussion

### Synthesis and Characterization of pH/ROS Cascade-Responsive VSeM-N=CH-PEG

The synthetic route of both VES-Se-Se-MTX and VES-N=CH-PEG as two key assembly motifs of VSeM-N=CH-PEG was illustrated in Scheme 1a. Typically, the VES-Se-Se-MTX was synthesized between VES and MTX via ROS-liable di-selenium linkage (Fig. S2), and VES-N=CH-PEG was synthesized between VES and PEG via tumor acidity-cleavable benzoic-imine linkage (Fig. S3). Then, the VSeM-N=CH-PEG nano-prodrug was prepared via di-selenium bridge-induced assembly of VES-Se-Se-MTX followed by surface insertion of VES-N=CH-PEG (Scheme 1b). The ^1^H nuclear magnetic resonance (NMR), Fourier transform infrared spectroscopy (FT-IR), ultraviolet–visible (UV–Vis), matrix-assisted laser desorption/ionization time-of-flight mass (MALDI-TOS MS), and ultrahigh-resolution Fourier transform-ion cyclotron resonance mass spectrometry (FT-ICR MS) results validated the chemical structure of different intermediate products and confirmed the successful synthesis of both VES-Se-Se-MTX and VES-N=CH-PEG (Figs. S4–S9). The evident changes in aforementioned ^1^H NMR and FT-IR spectra upon the introduction of weakly acidity and H_2_O_2_ demonstrated their pH/ROS-responsive characteristics (Fig. S10) [42].

**Fig. 1:**
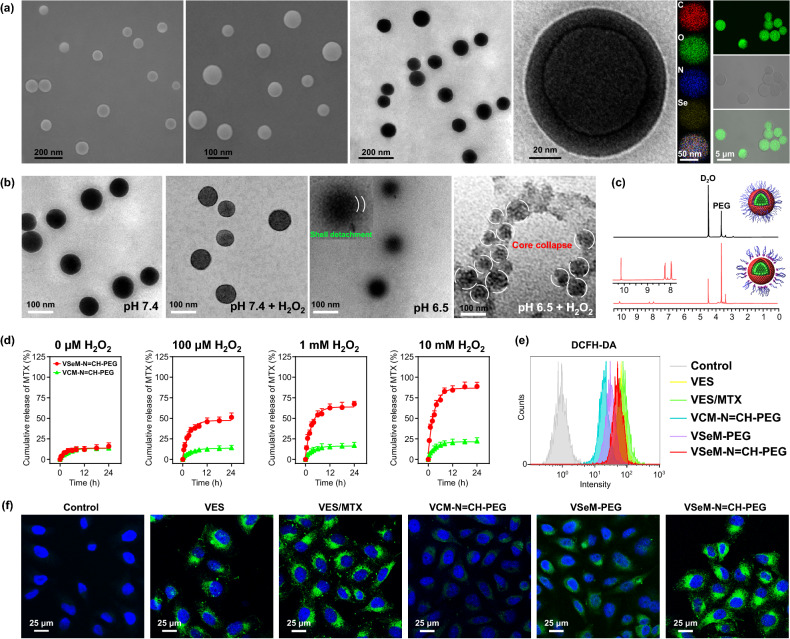
Characterization of VSeM-N=CH-PEG. **a** SEM, TEM, element mapping, and CLSM images of VSeM-N=CH-PEG nano-prodrug. **b** TEM images of VSeM-N=CH-PEG nano-prodrug at pH 7.4 and 6.5 without or with H_2_O_2_ (1 mM) for 0.5 h (pH 6.5) and 2 h (pH 7.4). **c** ^1^H NMR analysis of VSeM-N=CH-PEG nano-prodrug at pH 7.4 and 6.5. **d** Cumulative release of MTX from VSeM-N=CH-PEG at pH 6.5 with various concentrations of H_2_O_2_. **e** Flow cytometry profiles and **f** CLSM images of ROS levels in HeLa cells treated with VES/MTX, VCM-N=CH-PEG, VSeM-PEG, and VSeM-N=CH-PEG at pH 6.5 for 6 h. ROS was stained with DCFH-DA

The SEM, TEM, CLSM, and dynamic/electrophoretic light scattering (DLS/SLS) results revealed that the VSeM-N=CH-PEG exhibited a uniform spherical morphology, a hydrodynamic particle size of ~ 100 nm, and a negatively charged surface (Figs. 1a and S11a, b). The elemental mapping images clearly revealed the homogeneous distribution of four elements (C, N, O, and Se) in the assemblies (Fig. 1a). Furthermore, the VSeM-N=CH-PEG remained stable in various fluid circumstances including water, PBS, and PBS with 10% FBS for 5 days (Fig. S11c).

### Tumor Acidity-Responsive PEG Shell Detachment and ROS-Responsive VSeM Core Disassembly

To investigate the response of VSeM-N=CH-PEG to tumor acidity and ROS, TEM and DLS/SLS were used to analyze the morphology, size, and surface charge change of VSeM-N=CH-PEG before and after the stimuli of pH and ROS. As illustrated in Fig. 1b, the VSeM-N=CH-PEG exhibited no obvious shell detachment and core collapse in the absence of H_2_O_2_ at pH 7.4. Moreover, the outer PEG shell was partially or completely departed from the VSeM core and the PEG-detached VSeM could remain stable at pH 6.5. ^1^H NMR spectra, particle size, zeta potential change provided further evidences for the detachment of PEG shell around the VSeM core (Figs. 1c, S12, and S13).

Furthermore, the PEG-shielded VSeM core (pH 7.4) displayed no significant morphology change upon the addition of H_2_O_2_ within 2 h (Fig. 1b and S14a), whereas the PEG-detached VSeM core (pH 6.5) was partially disintegrated upon the addition of H_2_O_2_ (1 mM) within 0.5 h and nearly completely disintegrated within 2 h (Figs. 1b and S14b). The morphology observation was further supported by the size variations determined by DLS with similar trend (Fig. S14c). The underlying mechanism was that the further cleavage of di-selenium linkage triggered by ROS broke the hydrophobic/hydrophilic balance to result in the structural disintegration after dePEGylation caused by pH-triggered benzoic-imine cleavage [27, 43].

### In Vitro Drug Release

To further verify the ROS-responsive behaviors of VSeM-N=CH-PEG, the release profiles of MTX and VES from VSeM-N=CH-PEG were evaluated in PBS buffers with/without H_2_O_2_ at pH 6.5 [42], and the VCM-N=CH-PEG was employed as the control. Only ~ 15% of MTX and ~ 10% of VES were leaked from VSeM-N=CH-PEG at pH 6.5 in the absence of H_2_O_2_ for 24 h (Figs. 1d and S15). In contrast, approximately 50% of MTX and 40% of VES, respectively, released from VSeM-N=CH-PEG at pH 6.5 upon addition of 100 μM H_2_O_2_ (simulated tumor intracellular ROS environment). The faster release of MTX than VES was likely due to that the solubility of MTX in PBS was higher than that of VES. Furthermore, when the H_2_O_2_ level reached 10 mM, the cumulative release amount of MTX and VES increased to 90% and 70%, respectively. These results further demonstrated the triggering effect of H_2_O_2_ on drug release [44]. Additionally, the cumulative release of VSeM-N=CH-PEG was apparently faster compared with that of VCM-N=CH-PEG at different H_2_O_2_ levels. These results suggested the excellent structural stability of VSeM-N=CH-PEG in physiological conditions and its outstanding responsiveness to ROS for controlled drug release [45].

### In Vitro ROS Production

To explore the ROS generation efficiency of VSeM-N=CH-PEG in cancer cells, HeLa cells were incubated with VES, VES/MTX, VCM-N=CH-PEG, VSeM-PEG, and VSeM-N=CH-PEG in tumor acidic condition (pH 6.5), and then stained with 2′, 7′-dichlorofluorescein diacetate (DCFH-DA) as ROS indicator. As shown in Figs. 1e, f, and S16, VES, VES/MTX, and VSeM-N=CH-PEG produced significantly higher ROS compared with the control, VCM-N=CH-PEG, and VSeM-PEG group. It could be explained that the VSeM could be disassembled in response to intracellular ROS to trigger the release of VES, which could further produce ROS, and thus in turn accelerate the VSeM disassembly and VES release via positive-feedback loop. Furthermore, when HeLa cells were incubated with VSeM-N=CH-PEG at physiological pH, the evidently lower ROS levels were clearly observed (Fig. S17). This difference in ROS generation efficiency might be resulted from the difference in cellular internalization between pH 6.5 and 7.4 (discussed as below).

### In Vitro Cellular Uptake

**Fig. 2:**
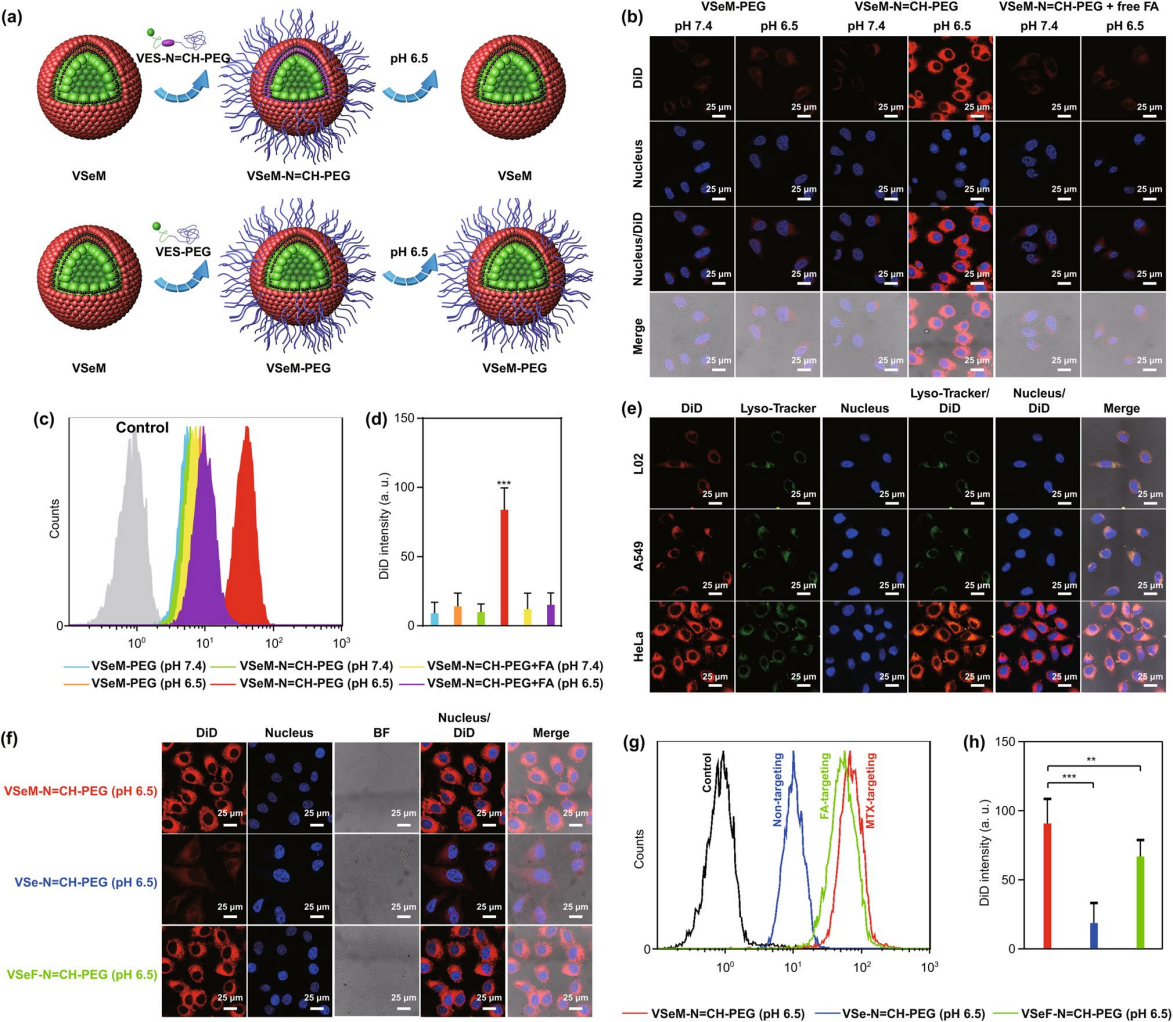
Tumor acidity-responsive active self-targeting recovery and cellular uptake. **a** Illustration of targeting activation of VSeM-N=CH-PEG and targeting inactivation of VSeM-PEG under tumor acidic condition. **b** CLSM images, **c** flow cytometry profiles, and **d** average fluorescence intensity of HeLa cells incubated with DiD-labeled VSeM-PEG, VSeM-N=CH-PEG, and VSeM-N=CH-PEG with pretreatment of free FA for 4 h. **e** CLSM images of L02, A549, HeLa cells incubated with DiD-labeled VSeM-PEG, VSeM-N=CH-PEG, and VSeM-N=CH-PEG with pretreatment of free FA at pH 6.5 for 4 h. **f** CLSM images, **g** flow cytometric profiles, and **h** average fluorescence intensity of HeLa cells incubated with VSe-N=CH-PEG, VSeF-N=CH-PEG, and VSeM-N=CH-PEG at pH 6.5 for 4 h. **, *P* < 0.01, ***, *P* < 0.005

To investigate the cellular uptake efficiency of VSeM-N=CH-PEG, HeLa cells with overexpressed folate receptors on the surface under physiological pH and tumor acidic extracellular pH, were evaluated by CLSM visualization and flow cytometry analysis. Prior to cell imaging, a lipophilic DiD fluorescence probe was encapsulated into VSeM-N=CH-PEG. As demonstrated in Fig. 2a–d and S18, the cellular uptake efficiency of VSeM-N=CH-PEG at pH 6.5 was approximately 8 times higher than that at pH 7.4. In contrast, the acidity-unresponsive VSeM-PEG (the similar morphology and size with VSeM-N=CH-PEG, Fig. S19) exhibited no distinctive difference in cellular uptake between pH 7.4 and 6.5, implying that the targeting ability was activated via the exposure of MTX ligands rather than pH variation. Moreover, the uptake of VSeM-N=CH-PEG in HeLa cells was dramatically reduced upon co-incubation with FA (a preferred ligand of folate receptor) at pH 6.5 (Fig. 2b–d); the internalization of VSeM-N=CH-PEG into A549 cells (a human lung cancer cell line) or L02 cells (a normal human liver cell line) at pH 6.5 was also evidently lower compared to that in HeLa cells at the same pH, because of the extremely low expression of folate receptors on A549 or L02 cells (Fig. 2e). These results consistently demonstrated that VSeM-N=CH-PEG possessed excellent tumor acidity-responsive targeting recovery capability [46]. In addition, the VSeM-N=CH-PEG was comparable in cellular uptake efficiency to VSeF-N=CH-PEG (VES-Se-Se-MTX was replaced with VES-Se-Se-FA, Fig. S20) under tumor acidic extracellular pH (Fig. 2f–h). These results demonstrated the high targeting efficiency of PEG shell-detached VSeM core.

### Macrophage Clearance

Owing to the immunogenic, hydrophobic, and exogenous property of targeting ligands, the delivery efficiency to tumor sites could be inevitably limited by macrophage recognition and clearance [47, 48]. Thus, the uptake of VSeM-N=CH-PEG by macrophages was investigated on RAW 264.7 cells at physiological pH. The VSeM without dynamic PEGylation was used as the control. As shown in Fig. S21, the uptake efficiency of VSeM-N=CH-PEG by RAW 264.7 cells was evidently decreased compared with that of VSeM, due to that the PEG shielding could effectively decrease the macrophage capture. Thus, the VSeM-N=CH-PEG could evade immune clearance by macrophages, which would be helpful to prolong circulation time and increase tumor accumulation [19, 47].

### In Vitro Anti-tumor Activity

**Fig. 3:**
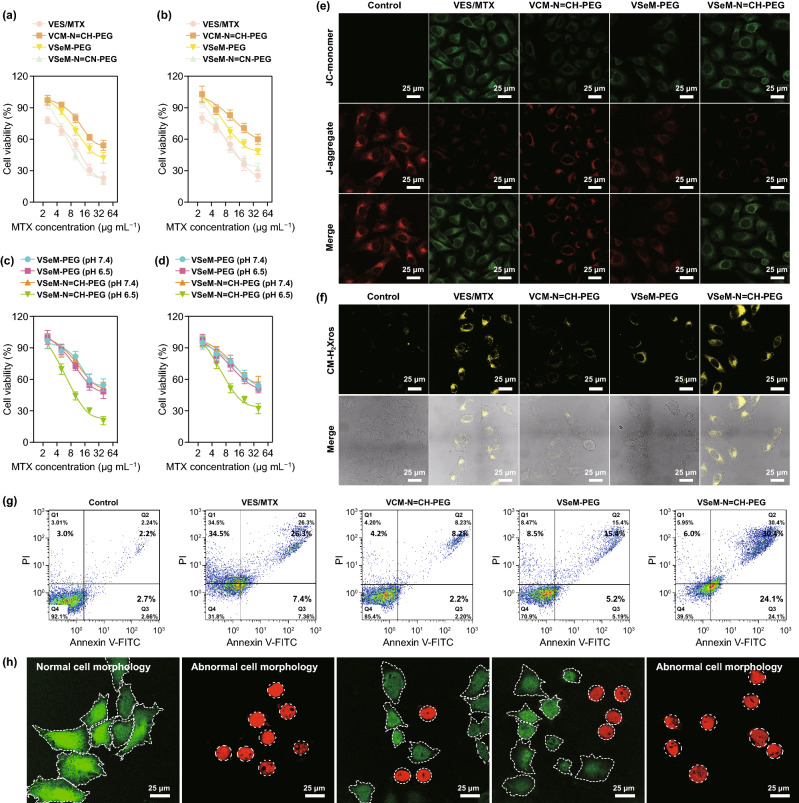
In vitro anti-tumor activity. **a** Viability of HeLa and **b** MCF-7 cells treated with VES/MTX, VCM-N=CH-PEG, VSeM-PEG, and VSeM-N=CH-PEG at pH 6.5 for 24 h. **c** Viability of HeLa and **d** MCF-7 cells treated with VSeM-PEG and VSeM-N=CH-PEG at pH 7.4 and 6.5 for 24 h. **e** Mitochondrial depolarization using JC-1 and **f** cellular oxidative stress using MitoTracker Red CM-H_2_Xros in HeLa cells treated with VES/MTX, VCM-N=CH-PEG, VSeM-PEG, and VSeM-N=CH-PEG at pH 6.5 for 12 h. **g** Apoptosis via Annexin V-FITC/PI assay and **h** live-dead cell staining via Calcein-AM/PI assay of HeLa cells induced by VES/MTX, VCM-N=CH-PEG, VSeM-PEG, and VSeM-N=CH-PEG after 12 h of incubation at pH 6.5

To investigate the anti-tumor activity of VSeM-N=CH-PEG, the cytotoxicity of different formulations toward HeLa and MCF-7 cells was firstly examined using a standard MTT assay. Prior to cell experiments, the pH of cell culture medium was adjusted to 6.5. As shown in Fig. 3a, b, the VSeM-N=CH-PEG treatment led to similar cytotoxicity to VES/MTX. Moreover, the VSeM-N=CH-PEG displayed remarkably improved cytotoxic damages against cancer cells compared with VCM-N=CH-PEG [24].

Additionally, more than 80% of normal cells including HUVEC and L02 cells survived from VSeM-N=CH-PEG treatment at physiological pH in the same concentration range (Fig. S22), implying the negligible cytotoxicity of VSeM-N=CH-PEG toward normal cells. These results revealed that VSeM-N=CH-PEG could efficiently and selectively kill cancer cells while keeping no harm to normal cells.

We further try to clarify the cytotoxicity of VSeM-N=CH-PEG toward cancer cells at various pH conditions (7.4 and 6.5). As shown in Fig. 3c, d, the VSeM-PEG exhibited similar level of cytotoxic damage at both acidic and physiological pH conditions. On the contrary, the cytotoxic damage of VSeM-N=CH-PEG at tumor acidic pH was significantly higher than that at physiological pH. This substantial increase in cytotoxicity proved the tumor acidity-responsive active targeting recovery. Besides, the FA pre-blocking dramatically reduced the cytotoxicity of VSeM-N=CH-PEG against HeLa cells at pH 6.5 due to the less effective cellular uptake (Fig. S23). These results validated the folate receptor-mediated endocytosis of VSeM-N=CH-PEG upon acidity-triggered PEG shell detachment [49, 50].

To further clarify intracellular changes induced by VSeM-N=CH-PEG, the mitochondrial membrane potential decrease and mitochondrial oxidative stress state, were investigated using a JC-1 fluorescent probe and a MitoTracker Red CM-H_2_Xros fluorescent probe, respectively (Fig. 3e, f) [51]. Compared with the VCM-N=CH-PEG and VSeM-PEG-treated HeLa cells, the VSeM-N=CH-PEG-treated cells exhibited a significant decrease in mitochondrial membrane potential, as revealed by the increased green fluorescence from JC-1 monomer and decreased red fluorescence from J-aggregate (Figs. 3e, S24a, and S25). Moreover, the VSeM-N=CH-PEG-treated HeLa cells induced a significant increase in mitochondrial oxidative stress state, as reflected by the enhanced orange-yellow fluorescence of the oxidized species which was converted from MitoTracker Red CM-H_2_Xros by mitochondrial ROS-induced oxidization (Figs. 3f and S24b). Furthermore, the VSeM-N=CH-PEG also starkly upregulated intracellular ROS levels and caspase-9/caspase-3 activity, and downregulated intracellular ATP levels (Fig. S26). Overall, our VSeM-N=CH-PEG could effectively blockATP supply and induce programmed cell death by mitochondrial dysfunction [51, 52].

The cytotoxicity data and intracellular changes were further supported by calcein AM/PI-mediated live/dead cell staining assay as well as cell morphology analysis (Fig. 3g). Moreover, the Annexin V-FITC/PI apoptosis analysis revealed that the VSeM-N=CH-PEG induced the most severe apoptosis among all formulations (Fig. 3h and S27). Thereby, the enhanced anti-tumor activity could be explained by the tumor acidity-triggered active targeting recovery, the amplified ROS-triggered drug release, and the disruption of mitochondrial functions [53].

### In Vivo Pharmaceutics and Biodistribution

Prior to in vivo study, blood hemolysis test was carried out to study the blood compatibility of VSeM-N=CH-PEG. No obvious hemolysis was observed from blood treated with VSeM-N=CH-PEG at the highest concentration of ~ 1 mg mL^−1^ for 4 h (Fig. S28), suggesting the minimum damage of VSeM-N=CH-PEG against red blood cells and their excellent blood biocompatibility.

**Fig. 4:**
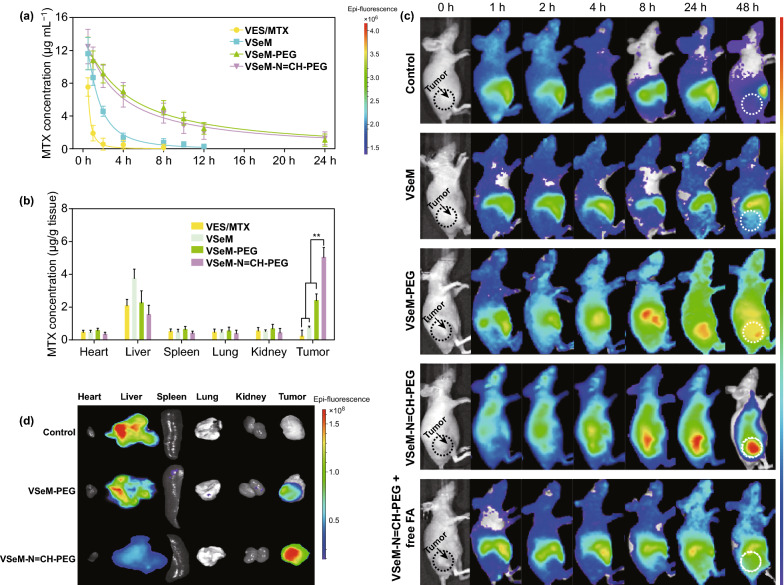
In vivo pharmacokinetics, biodistribution, and tumor accumulation of VSeM-N=CH-PEG. **a** Pharmacokinetics and **b** biodistribution of HeLa tumor-bearing mice after intravenous injection of DiR-labeled VES/MTX, VSeM, VSeM-PEG, and VSeM-N=CH-PEG. DiR was used as a control. **c** Time-lapsed NIR fluorescence imaging of HeLa tumor-bearing mice. **d** NIR fluorescence imaging of excised normal and tumor tissues at 48 h post-injection. Error bars indicate SD (*n* = 3). **P < 0.01

To monitor the in vivo pharmacokinetics of VSeM-N=CH-PEG, the plasma drug concentration was determined following intravenous injection to HeLa tumor-bearing nude mice. As exhibited in Fig. 4a, the residual amount of VSeM-N=CH-PEG and VSeM-PEG in the bloodstream decayed much slower than that of VSeM and even VES/MTX, indicating the elongated circulation time of VSeM-N=CH-PEG.

Considering the increased circulation longevity would enhance the accumulation of drug in tumors via both EPR effect and ligand-mediated active targeting, we subsequently investigated the biodistribution of VSeM-N=CH-PEG in vivo. As shown in Fig. 4b, the drug amount of VSeM-N=CH-PEG accumulated at tumor site was remarkably higher compared with that of VSeM-PEG, VSeM, and VES/MTX. Moreover, the amount of drug delivered by VSeM-N=CH-PEG to tumor tissues was remarkably higher than that delivered to the other organs. These results revealed that the VSeM-N=CH-PEG could deliver drugs to tumors via both EPR effect-based passive targeting and tumor acidity-activated active targeting mechanisms [54].

### In Vivo Tumor Accumulation

To investigate the accumulation ability of VSeM-N=CH-PEG in tumors, fluorescence imaging was performed on HeLa tumor-bearing nude mice. For animal imaging, DiR, a lipophilic near-infrared (NIR) probe, was encapsulated within VSeM-N=CH-PEG. As illustrated in Figs. 4c, S29, and S30a, the fluorescence signals of VSeM-PEG in tumors reached a peak at 24 h post-injection; thereafter, the fluorescence signals began to decline. By sharp contrast, the fluorescence signals of VSeM-N=CH-PEG in tumors continued to increase until 48 h, and the fluorescence signals were significantly and consistently stronger than that of VSeM-PEG-injected mice over a period of 48 h. This result demonstrated both superior tumor accumulation and stronger tumor cell internalization of VSeM-N=CH-PEG, and this conclusion could be further supported by the result of ex vivo fluorescence imaging (Figs. 4d and S30b) and CLSM imaging of frozen sections (Fig. S30c). Moreover, the VSeM-N=CH-PEG-injected mice with FA pre-infusion exhibited significantly lower tumor accumulation than those without FA pre-infusion, indicating a key role of folate receptor-dependent cellular uptake in vivo. In addition, the accumulation of VSeM-N=CH-PEG in liver was lower than that of VSeM-PEG and VSeM, demonstrating the reduced immune recognition and reticuloendothelial system (RES) capture of VSeM-N=CH-PEG. Taken together, these results demonstrated the outstanding tumor accumulation and prolonged tumor retention of VSeM-N=CH-PEG.

### In Vivo Anti-tumor Efficacy of Oxidation-Chemotherapy

**Fig. 5:**
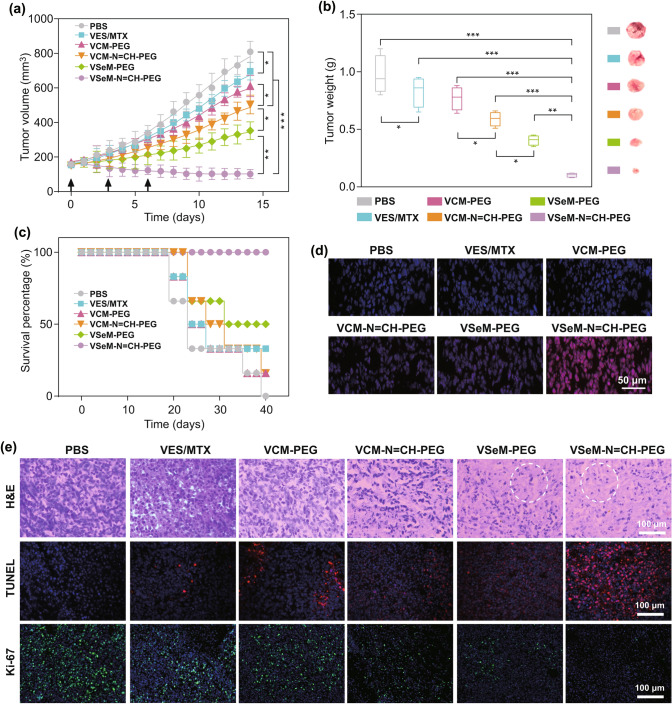
In vivo anti-tumor effects of VSeM-N=CH-PEG. **a** Tumor growth profiles of HeLa tumor-bearing nude mice after different treatments with PBS, VES/MTX, VCM-PEG, VCM-N=CH-PEG, VSeM-PEG, and VSeM-N=CH-PEG at equivalent MTX concentration (4 mg kg^−1^) during 14 days. **b** Tumor weight excised from HeLa tumor-bearing nude mice on the 14th day. **c** Survival percentage of HeLa tumor-bearing nude mice after different treatments. **d** Cellular ROS levels in tumor excised from mice by dihydroethidium (DHE) staining on the 2nd day. **e** Representative images of H&E, TUNEL, and Ki-67-stained tumor sections, respectively. White circles indicated cell apoptosis. *P < 0.05, **P < 0.01, ***P < 0.001

Encouraged by the excellent tumor accumulation, we investigated the anti-tumor efficacy of VSeM-N=CH-PEG in vivo. As illustrated in Figs. 5a and S31a, the groups treated with PBS exhibited remarkably rapid tumor size increase. Besides, the groups receiving treatment with VES/MTX showed a slight suppression of tumor growth due to the very limited accumulation of free VES and MTX in tumors. In contrast, the groups receiving treatment with VCM-N=CH-PEG and VSeM-PEG showed better tumor growth inhibition because of active targeting recovery or ROS generation, respectively. But above all, the groups receiving treatment with VSeM-N=CH-PEG exhibited the strongest anti-tumor efficacy that the tumor size was evidently decreased after treatment for 14 days without any body weight loss (Fig. S31b). The above results were again supported by the analysis of excised tumor weight/volume (Figs. 5b and S31c) and the tumor inhibition rate (Fig. S31d). Additionally, the VSeM-N=CH-PEG group also remarkably extend the survival time of tumor-bearing mice in comparison with the other treatment groups after 40 days (Fig. 5c). The reasons for the remarkably increased anti-tumor efficiency could be elucidated as follows: (1) PEG shielding effect of VSeM-N=CH-PEG could increase the circulation longevity and enhance the tumor accumulation via EPR effect; (2) tumor acidity-activated self-targeting could effectively increase internalization efficiency of VSeM-N=CH-PEG; (3) intracellular ROS could trigger structure collapse and drug release, and released VES would further generate ROS (Fig. 5d) for amplification of drug release; (4) VES could not only inhibit ATP production but also synergize with MTX, provoking tumor cell apoptosis with high-efficiency. The integration of active self-targeting recovery and ROS amplification strategy contributed to the pronounced therapeutic effect of VSeM-N=CH-PEG.

Hematoxylin-eosin (H&E), TUNEL, and Ki 67 staining assay were carried out to further verify the improved anti-tumor effect of VSeM-N=CH-PEG (Fig. 5e). Among all groups, the VSeM-N=CH-PEG groups resulted in the most significant cell apoptosis activity, as observed by the most severe cellular shrinkage and nuclear condensation in H&E staining images, and the most extensive apoptotic nuclear fragmentation in TUNEL staining images, and the lowest Ki-67 expression in Ki-67 staining images. These results revealed that the VSeM-N=CH-PEG could efficiently kill tumor cells in vivo.

### In Vivo Biosafety

The biosafety of VSeM-N=CH-PEG nano-prodrug, a prerequisite for their in vivo applications, was further investigated by histological, hematological, and blood biochemical analysis. Negligible morphological/histological damage in major organs (Fig. S32), no significant change in hematological/biochemical indexes (Figs. S33 and S34), and no body weight loss were found in VSeM-N=CH-PEG groups, indicating the superior in vivo biocompatibility of VSeM-N=CH-PEG. The results consistently revealed that the VSeM-N=CH-PEG nano-prodrug significantly enhanced anti-tumor efficiency while reduced the systematic toxicity.

## Conclusions

In summary, a cascade-triggered programmable anticancer nano-prodrug with self-targeting-activation and ROS amplification was proposed for highly efficient synergistic tumor therapy. Both in vitro and in vivo studies comprehensively validated that the VSeM-N=CH-PEG not only exhibited better tumor accumulation, prolonged tumor retention, and enhanced cellular uptake via tumor acidity-triggered active self-targeting, but also efficiently generated extra ROS to achieve accelerated structure collapse and drug release while blocking ATP supply. Taken together, here proposed strategy shows dramatically improved therapeutic effect with negligible toxicity via the synergy between oxidation therapy and chemotherapy, and it will inspire the development of safe and effective drug delivery systems in future.

## Electronic supplementary material

Below is the link to the electronic supplementary material.

Experimental section and additional characterization/bioapplication data for VSeM-N**=**CH-PEG including Fig. S1-S34 (PDF 3394 kb)

## References

[1] Wang D, Yu C, Xu L, Shi L, Tong G (2018). Nucleoside analogue-based supramolecular nanodrugs driven by molecular recognition for synergistic cancer therapy. J. Am. Chem. Soc..

[2] Xin X, Du X, Xiao Q, Azevedo HS, He W, Yin L (2019). Drug nanorod-mediated intracellular delivery of microrna-101 for self-sensitization via autophagy inhibition. Nano-Micro Lett..

[3] Hou J, Pan Y, Zhu D, Fan Y, Feng G (2019). Targeted delivery of nitric oxide via a ‘bump-and-hole’’-based enzyme-prodrug pair. Nat. Chem. Biol..

[4] Ekladious I, Colson YL, Grinstaff MW (2019). Polymer-drug conjugate therapeutics: advances, insights and prospects. Nat. Rev. Drug Discov..

[5] Xu S, Zhu X, Huang W, Zhou Y, Yan D (2017). Supramolecular cisplatin-vorinostat nanodrug for overcoming drug resistance in cancer synergistic therapy. J. Control. Release.

[6] Liang X, Gao C, Cui L, Wang S, Wang J, Dai Z (2017). Self-assembly of an amphiphilic janus camptothecin-floxuridine conjugate into liposome-like nanocapsules for more efficacious combination chemotherapy in cancer. Adv. Mater..

[7] Huang P, Wang D, Su Y, Huang W, Zhou Y (2014). Combination of small molecule prodrug and nanodrug delivery: amphiphilic drug-drug conjugate for cancer therapy. J. Am. Chem. Soc..

[8] Shi J, Kantoff PW, Wooster R, Farokhzad OC (2017). Cancer nanomedicine: progress, challenges and opportunities. Nat. Rev. Cancer.

[9] Yongvongsoontorn N, Chung JE, Gao SJ, Bae KH, Yamashita A (2019). Carrier-enhanced anticancer efficacy of sunitinib-loaded green tea-based micellar nanocomplex beyond tumor-targeted delivery. ACS Nano.

[10] Wang S, Huang P, Chen X (2016). Hierarchical targeting strategy for enhanced tumor tissue accumulation/retention and cellular internalization. Adv. Mater..

[11] Wang S, Yu G, Wang Z, Jacobson O, Tian R (2018). Hierarchical tumor microenvironment-responsive nanomedicine for programmed delivery of chemotherapeutics. Adv. Mater..

[12] El-Sawy HS, Al-Abd AM, Ahmed TA, El-Say KM, Torchilin VP (2018). Stimuli-responsive nano-architecture drug-delivery systems to solid tumor micromilieu: past, present, and future perspectives. ACS Nano.

[13] Li M, Sun X, Zhang N, Wang W, Yang Y, Jia H, Liu W (2018). Nir-activated polydopamine-coated carrier-free “nanobomb” for in situ on-demand drug release. Adv. Sci..

[14] Li X, Liu Y, Fu F, Cheng M, Liu Y (2019). Single nir laser-activated multifunctional nanoparticles for cascaded photothermal and oxygen-independent photodynamic therapy. Nano-Micro Lett..

[15] Castillo RR, Lozano D, Gonzalez B, Manzano M, Izquierdo-Barba I, Vallet-Regi M (2019). Advances in mesoporous silica nanoparticles for targeted stimuli-responsive drug delivery: an update. Exp. Opin. Drug Deliv..

[16] Du J, Lane LA, Nie S (2015). Stimuli-responsive nanoparticles for targeting the tumor microenvironment. J. Control. Release.

[17] Cui M, Liu S, Song B, Guo D, Wang J (2019). Fluorescent silicon nanorods-based nanotheranostic agents for multimodal imaging-guided photothermal therapy. Nano-Micro Lett..

[18] Jiang Z, Yuan B, Qiu N, Wang Y, Sun L (2019). Manganese-zeolitic imidazolate frameworks-90 with high blood circulation stability for mri-guided tumor therapy. Nano-Micro Lett..

[19] Jin Q, Deng Y, Chen X, Ji J (2019). Rational design of cancer nanomedicine for simultaneous stealth surface and enhanced cellular uptake. ACS Nano.

[20] Mukerabigwi JF, Yin W, Zha Z, Ke W, Wang Y (2019). Polymersome nanoreactors with tumor ph-triggered selective membrane permeability for prodrug delivery, activation, and combined oxidation-chemotherapy. J. Control. Release.

[21] Lu J, Wang J, Ling D (2018). Surface engineering of nanoparticles for targeted delivery to hepatocellular carcinoma. Small.

[22] Davoodi P, Lee LY, Xu Q, Sunil V, Sun Y, Soh S, Wang CH (2018). Drug delivery systems for programmed and on-demand release. Adv. Drug Deliv. Rev..

[23] Yang GG, Zhang H, Zhang DY, Cao Q, Yang J, Ji LN, Mao ZW (2018). Cancer-specific chemotherapeutic strategy based on the vitamin k3 mediated ros regenerative feedback and visualized drug release in vivo. Biomaterials.

[24] Xu X, Saw PE, Tao W, Li Y, Ji X (2017). Ros-responsive polyprodrug nanoparticles for triggered drug delivery and effective cancer therapy. Adv. Mater..

[25] Lv X, Zhu Y, Ghandehari H, Yu A, Wang Y (2019). An ros-responsive and self-accelerating drug release nanoplatform for overcoming multidrug resistance. Chem. Commun..

[26] Saravanakumar G, Kim J, Kim WJ (2017). Reactive-oxygen-species-responsive drug delivery systems: promises and challenges. Adv. Sci..

[27] Dai L, Li X, Duan X, Li M, Niu P (2019). A ph/ros cascade-responsive charge-reversal nanosystem with self-amplified drug release for synergistic oxidation-chemotherapy. Adv. Sci..

[28] Broaders KE, Grandhe S, Fréchet JMJ (2011). A biocompatible oxidation-triggered carrier polymer with potential in therapeutics. J. Am. Chem. Soc..

[29] de Gracia Lux C, Joshi-Barr S, Nguyen T, Mahmoud E, Schopf E, Fomina N, Almutairi A (2012). Biocompatible polymeric nanoparticles degrade and release cargo in response to biologically relevant levels of hydrogen peroxide. J. Am. Chem. Soc..

[30] Schnipper L (1986). Clinical implications of tumor-cell heterogeneity. N. Engl. J. Med..

[31] Dong LF, Low P, Dyason JC, Wang XF, Prochazka L (2008). Α-tocopheryl succinate induces apoptosis by targeting ubiquinone-binding sites in mitochondrial respiratory complex II. Oncogene.

[32] Neuzil J (2003). Vitamin e succinate and cancer treatment: a vitamin e prototype for selective antitumour activity. Br. J. Cancer.

[33] Dong LF, Jameson VJ, Tilly D, Cerny J, Mahdavian E (2011). Mitochondrial targeting of vitamin e succinate enhances its pro-apoptotic and anti-cancer activity via mitochondrial complex II. J. Biol. Chem..

[34] Weber T, Lu M, Andera L, Lahm H, Gellert N (2002). Vitamin E succinate is a potent novel antineoplastic agent with high selectivity and cooperativity with tumor necrosis factor-related apoptosis-inducing ligand (apo2 ligand) in vivo. Clin. Cancer Res..

[35] Dong L-F, Freeman R, Liu J, Zobalova R, Marin-Hernandez A (2009). Suppression of tumor growth in vivo by the mitocan α-tocopheryl succinate requires respiratory complex II. Clin. Cancer Res..

[36] Dong LF, Swettenham E, Eliasson J, Wang XF, Gold M (2007). Vitamin E analogues inhibit angiogenesis by selective induction of apoptosis in proliferating endothelial cells: the role of oxidative stress. Cancer Res..

[37] Song XR, Li SH, Guo H, You W, Tu D (2018). Enhancing antitumor efficacy by simultaneous atp-responsive chemodrug release and cancer cell sensitization based on a smart nanoagent. Adv. Sci..

[38] Mizusawa K, Takaoka Y, Hamachi I (2012). Specific cell surface protein imaging by extended self-assembling fluorescent turn-on nanoprobes. J. Am. Chem. Soc..

[39] Li Y, Lin J, Cai Z, Wang P, Luo Q (2020). Tumor microenvironment-activated self-recognizing nanodrug through directly tailored assembly of small-molecules for targeted synergistic chemotherapy. J. Control. Release.

[40] Lin W, Sun T, Xie Z, Gu J, Jing X (2016). A dual-responsive nanocapsule via disulfide-induced self-assembly for therapeutic agent delivery. Chem. Sci..

[41] Wang Y, Liu D, Zheng Q, Zhao Q, Zhang H (2014). Disulfide bond bridge insertion turns hydrophobic anticancer prodrugs into self-assembled nanomedicines. Nano Lett..

[42] Pu HL, Chiang WL, Maiti B, Liao ZX, Ho YC (2014). Nanoparticles with dual responses to oxidative stress and reduced ph for drug release and anti-inflammatory applications. ACS Nano.

[43] Kamaly N, Yameen B, Wu J, Farokhzad OC (2016). Degradable controlled-release polymers and polymeric nanoparticles: mechanisms of controlling drug release. Chem. Rev..

[44] Shao D, Li M, Wang Z, Zheng X, Lao YH (2018). Bioinspired diselenide-bridged mesoporous silica nanoparticles for dual-responsive protein delivery. Adv. Mater..

[45] Liang K, Chung JE, Gao SJ, Yongvongsoontorn N, Kurisawa M (2018). Highly augmented drug loading and stability of micellar nanocomplexes composed of doxorubicin and poly(ethylene glycol)-green tea catechin conjugate for cancer therapy. Adv. Mater..

[46] Li Y, Lin J, Wang P, Luo Q, Lin H (2019). Tumor microenvironment responsive shape-reversal self-targeting virus-inspired nanodrug for imaging-guided near-infrared-II photothermal chemotherapy. ACS Nano.

[47] Walkey CD, Olsen JB, Guo H, Emili A, Chan WC (2012). Nanoparticle size and surface chemistry determine serum protein adsorption and macrophage uptake. J. Am. Chem. Soc..

[48] Lee SB, Lee J, Cho SJ, Chin J, Kim SK (2019). Crushed gold shell nanoparticles labeled with radioactive iodine as a theranostic nanoplatform for macrophage-mediated photothermal therapy. Nano-Micro Lett..

[49] Mi P, Cabral H, Kataoka K (2019). Ligand-installed nanocarriers toward precision therapy. Adv. Mater..

[50] Wang S, Dormidontova EE (2012). Selectivity of ligand-receptor interactions between nanoparticle and cell surfaces. Phys. Rev. Lett..

[51] Guan Q, Zhou LL, Li YA, Li WY, Wang S, Song C, Dong YB (2019). Nanoscale covalent organic framework for combinatorial antitumor photodynamic and photothermal therapy. ACS Nano.

[52] Tuguntaev RG, Chen S, Eltahan AS, Mozhi A, Jin S (2017). P-gp inhibition and mitochondrial impairment by dual-functional nanostructure based on vitamin e derivatives to overcome multidrug resistance. ACS Appl. Mater. Interfaces..

[53] Wen R, Banik B, Pathak RK, Kumar A, Kolishetti N, Dhar S (2016). Nanotechnology inspired tools for mitochondrial dysfunction related diseases. Adv. Drug Deliv. Rev..

[54] Wilhelm S, Tavares AJ, Dai Q, Ohta S, Audet J, Dvorak HF, Chan WCW (2016). Analysis of nanoparticle delivery to tumours. Nat. Rev. Mater..

