# Supplementary material for: Tumor Microenvironment Cascade-Responsive Nanodrug with Self-Targeting Activation and ROS Regeneration for Synergistic Oxidation-Chemotherapy
Source: Nanomicro Lett. 2020 Sep 14;12:182. doi: 10.1007/s40820-020-00492-4 (PMC7770705; doi:10.1007/s40820-020-00492-4)
Supplement: Supplementary file 1 — Experimental section and additional characterization/bioapplication data for VSeM-N=CH-PEG including Fig. S1-S34 (PDF 3394 kb) [file 40820_2020_492_MOESM1_ESM.pdf]

Supporting Information for

Tumor Microenvironment Cascade-Responsive Nanodrug with Self-Targeting Activation and ROS Regeneration for Synergistic Oxidation-Chemotherapy

Yang Li[1, 2, 3, #], Jinyan Lin[2, #], Peiyuan Wang[1, 2, 3], Qiang Luo[1, 2, 3], Fukai Zhu[4], Yun Zhang[1, 3], Zhenqing Hou[4], Xiaolong Liu [1, 2, 3,*], Jingfeng Liu, [1, 2, 3,*]

[1]CAS Key Laboratory of Design and Assembly of Functional Nanostructures, Fujian Institute of Research on the Structure of Matter, Chinese Academy of Sciences, Fuzhou 350002, People's Republic of China

[2]The United Innovation of Mengchao Hepatobiliary Technology Key Laboratory of Fujian Province, Mengchao Hepatobiliary Hospital of Fujian Medical University, Fuzhou 350025, People's Republic of China

[3]Department of Translational Medicine, Xiamen Institute of Rare Earth Materials, Chinese Academy of Sciences, Xiamen 361024, People's Republic of China

[4]Xiamen Cardiovascular Hospital & College of Materials, Xiamen University, Xiamen 361005, People's Republic of China

[#]Y. Li and J. Lin contributed equally to this work

*Corresponding authors. E-mail: drjingfeng@126.com (Jingfeng Liu); Pof. xiaoloong.liu@gmail.com (Xiaolong Liu)

Supplementary Figures

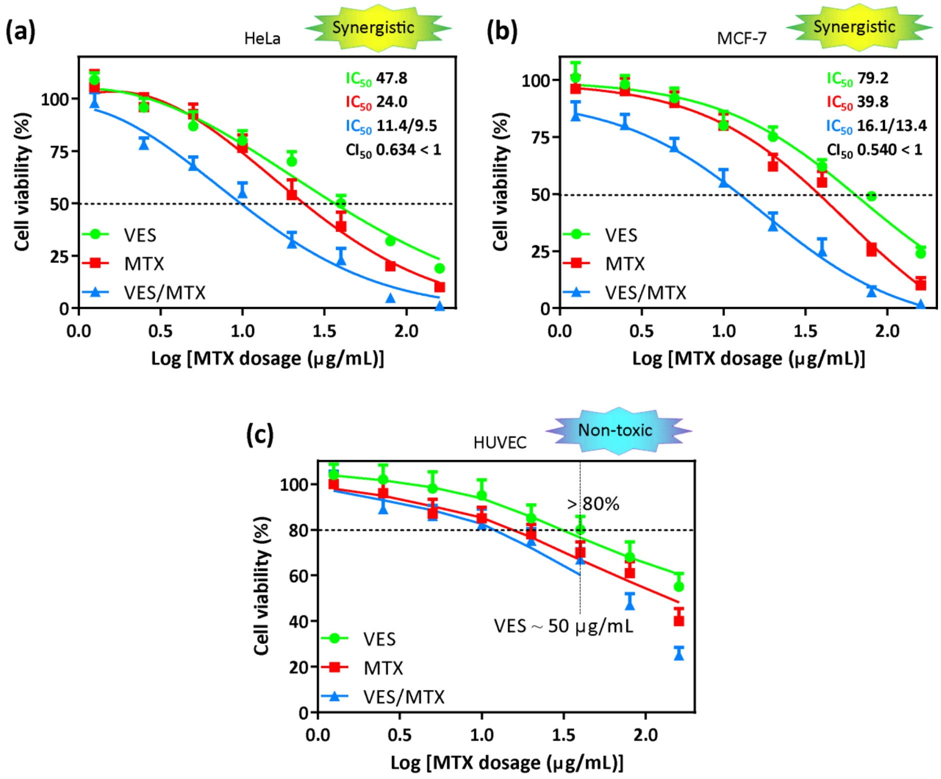

Fig. S1 Synergistic anti-tumor activity of VES/MTX against cancer cells (a, b) and non-toxicity of VES/MTX towards normal cells (c). The combination index (CI) was calculated with a classic isobologram equation of Chou-Talalay to determine whether the effects were additive (CI = 1), synergistic (CI < 1), or antagonistic (CI > 1).

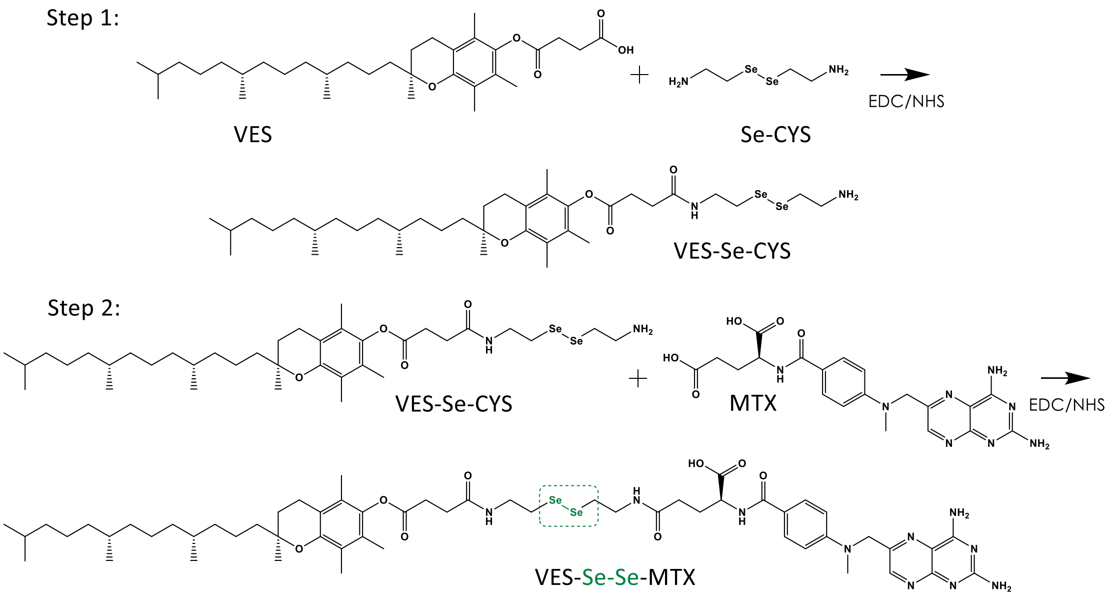

Fig. S2 Two-step synthesis of ROS-sensitive self-targeting prodrug VES-Se-Se-MTX

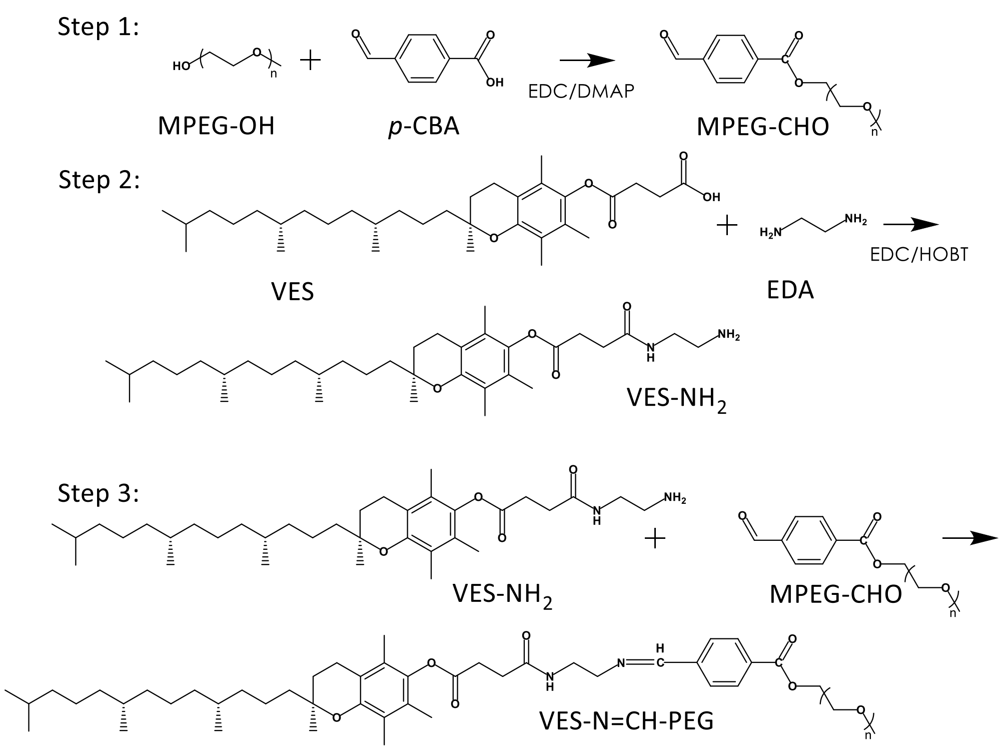

Fig. S3 Three-step synthesis of tumor acidity-sensitive VES-N=CH-PEG

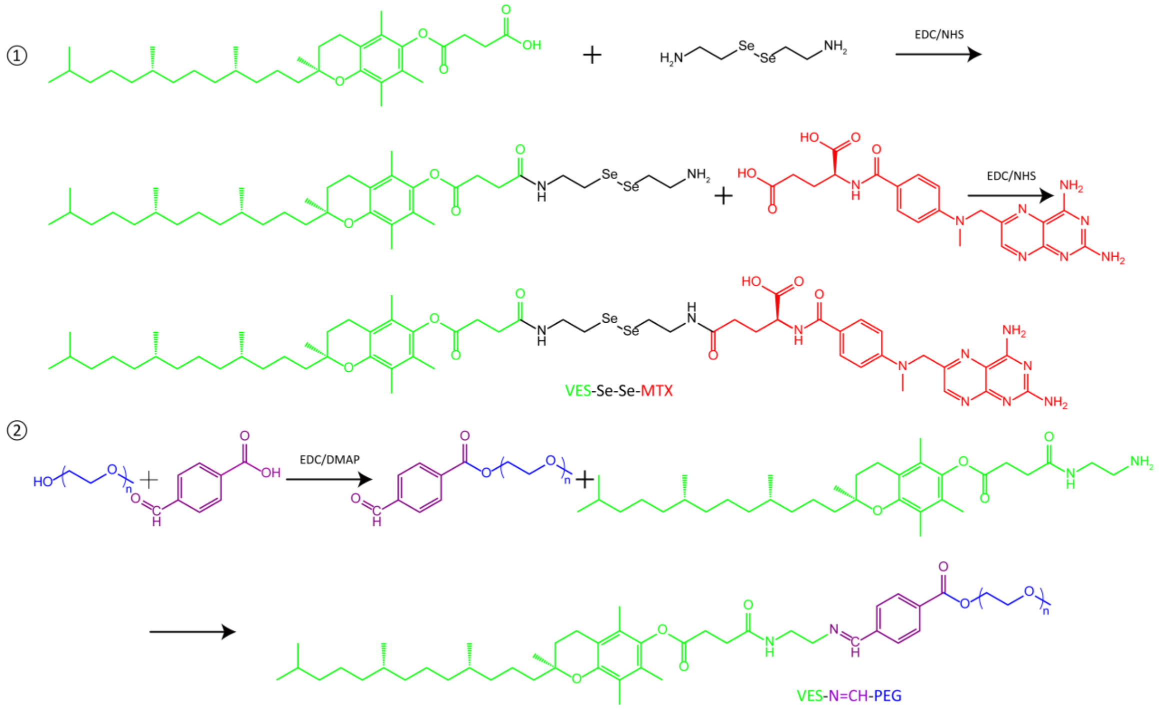

Fig. S4 Synthesis routes of ROS-responsive VES-Se-Se-MTX and tumor acidity-responsive VES-N=CH-PEG

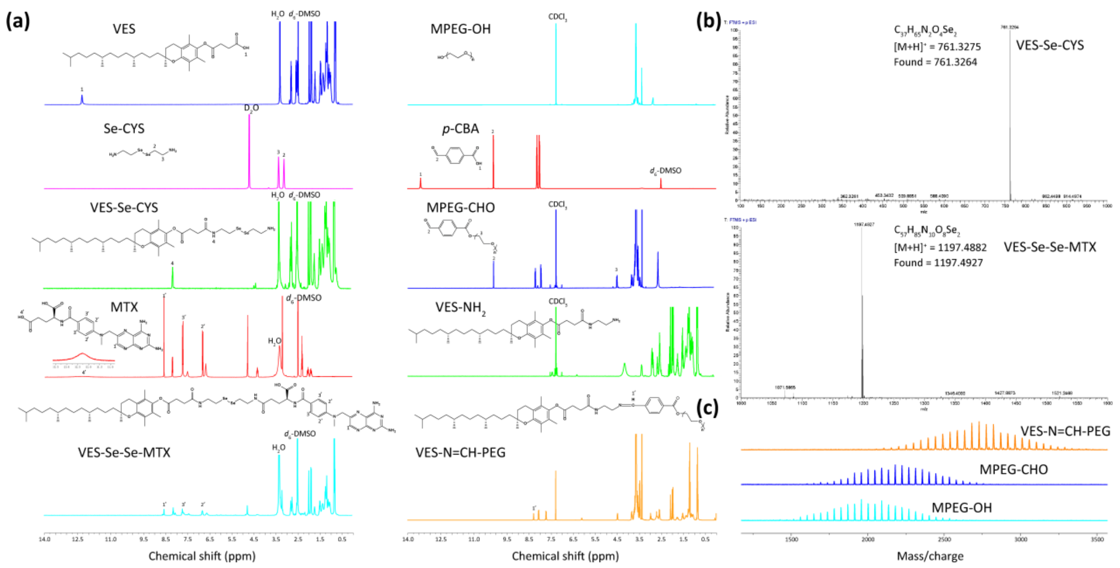

Fig. S5 Characterization of VES-Se-Se-MTX prodrug and VES-N=CH-PEG polymer. **a** ^{1}H NMR analysis of VES-Se-Se-MTX and VES-N=CH-PEG. **b** FT-MS analysis of VES-Se-Se-MTX. **c** MALDI-TOS-MS analysis of VES-N=CH-PEG

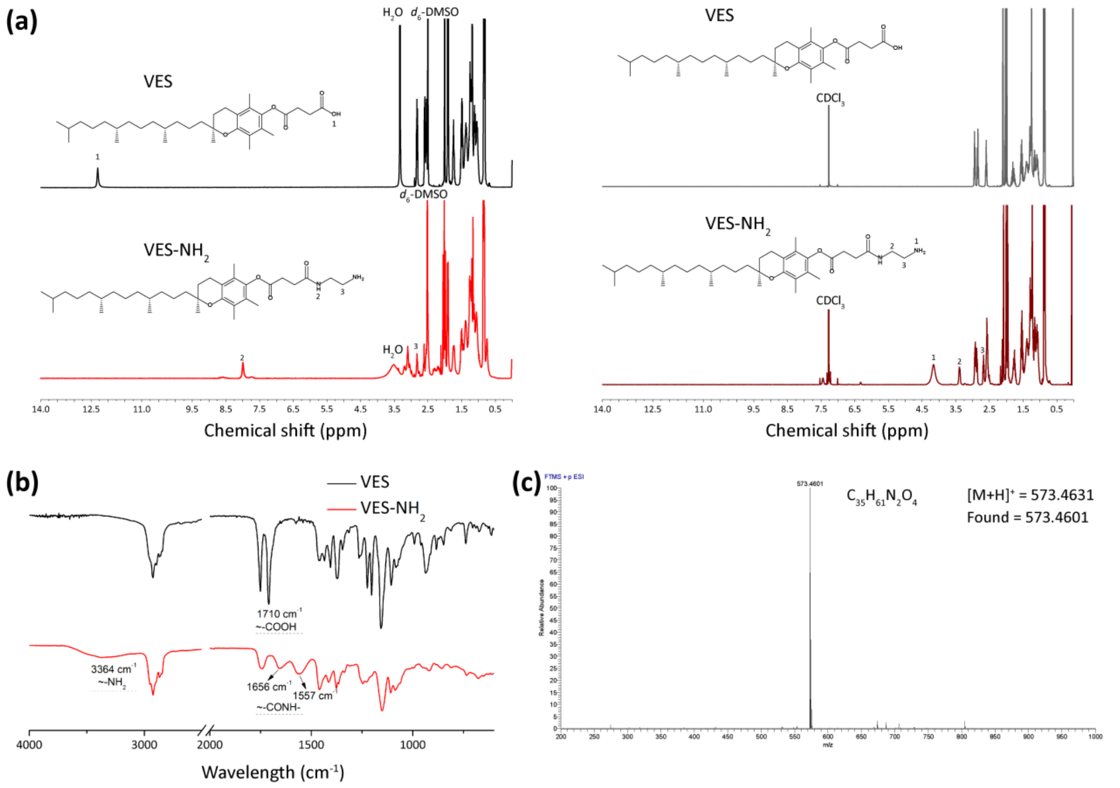

Fig. S6 a ^{1}H NMR spectra of VES and synthesized VES-NH_2. *d*$_6$-DMSO and $CDCl_3$ were employed as solvent, respectively. **b** FT-IR spectra of VES and synthesized VES-NH_2. **c** FT-MS spectrum of synthesized VES-NH_2

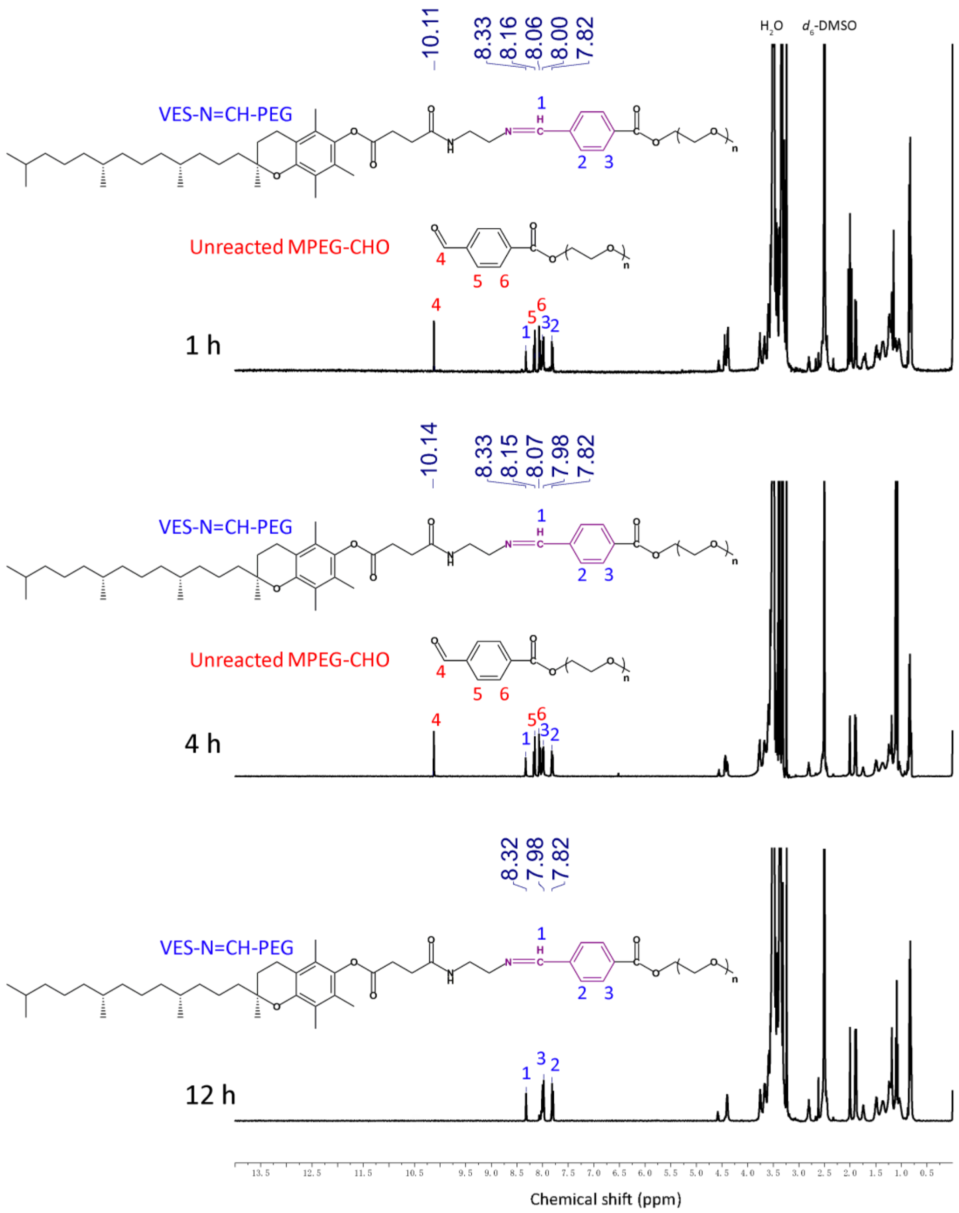

Fig. S7 Variation of ^{1}H NMR spectra of VES-N=CH-PEG after conjugation of VES-NH_2 and MPEG-CHO *via* benzoic-imine linkage for different reaction time periods

The aldehyde proton signal at ~10.1 ppm and aromatic proton signals at ~8.2 and ~8.1 ppm of unreacted MPEG-CHO gradually weaken and even completely disappeared after conjugation for 12 h, indicating completion of Schiff base reaction *via* benzoic-imine linkage.

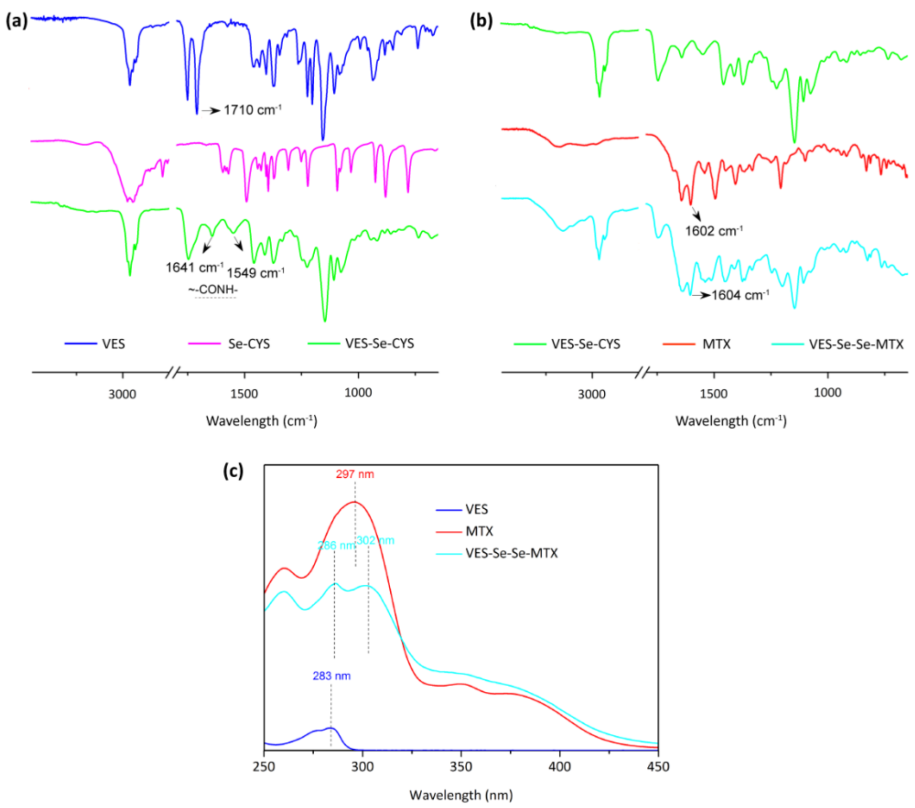

Fig. S8 a FT-IR spectra of VES, Se-CYS, and VES-Se-CYS. **b** FT-IR spectra of VES-Se-CYS, MTX, and VES-Se-Se-MTX. **c** UV-vis spectra of VES, MTX, and VES-Se-Se-MTX. DMSO was employed as solvent.

For FT-IR analysis, compared with the absorption peak in the spectrum of VES, the absorption peak at 1710 cm^{-1} derived from carbonyl (C=O) stretching vibration of VES carboxyl sharply decreased and even disappeared in the spectrum of VES-Se-CYS. Furthermore, two new absorption peaks appeared at 1641 and 1549 cm^{-1} in the spectrum of VES-Se-CYS, which was assigned to the stretching vibration of amide I (NC=O) and the bending vibration of amide II (CN-H), respectively. These results suggested the successful synthesis of VES-Se-CYS *via* the formation of amide bonds (HNC=O). After coupling VES-Se-CYS with MTX, an additional absorption peak clearly appeared at 1604 cm^{-1} in the spectrum of VES-Se-Se-MTX, which was originated from the N-H bending vibration of aromatic amine of MTX. These results suggested the successful coupling VES-Se-CYS with MTX. For UV-vis analysis, Compared to the observations for the characteristic absorption peaks of VES and MTX at 283 and 297 nm, respectively, a red shift in their absorption peaks of VES-Se-Se-MTX at 286 and 302 nm was respectively observed. The result could be attributed to successful introduction of a conjugated structure.

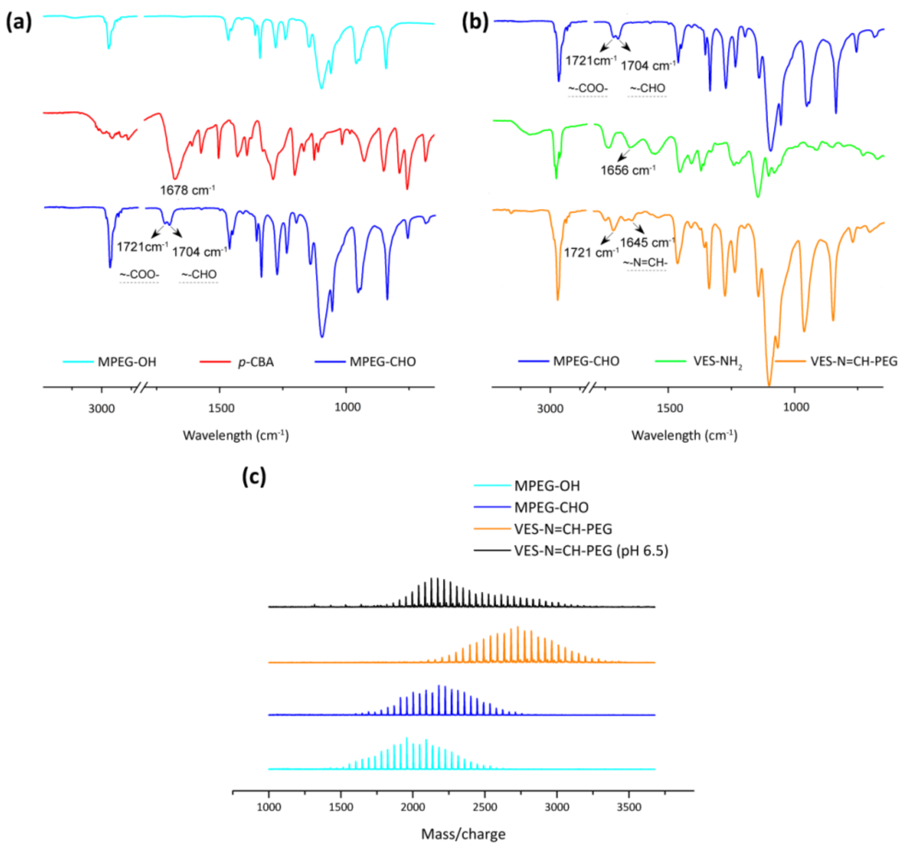

Fig. S9 a FT-IR spectra of MPEG-OH, *p*-CBA, MPEG-CHO, VES-NH_2, and VES-N=CH-PEG. **b** MALDI-TOF-MS spectra of MPEG-OH, MPEG-CHO, VES-N=CH-PEG, and VES-N=CH-PEG with addition of acid

A absorption peak at ~1704 cm^{-1} derived from the stretching vibration of aldehyde and another absorption peak at ~1721 cm^{-1} resulted from the newly formed ester linkage between MPEG-OH and aromatic carboxyl of *p*-CBA were evidently observed in the spectrum of MPEG-CHO. Further, a new absorption peak at 1645 cm^{-1} appeared and another absorption peak at 1704 cm^{-1} nearly disappeared after conjugating with VES-NH_2, indicating the formation of benzoic-imine bond (-N=CH-) in conjugate process. These results suggested the successful synthesis of VES-N=CH-PEG *via* the formation of benzoic-imine linkage.

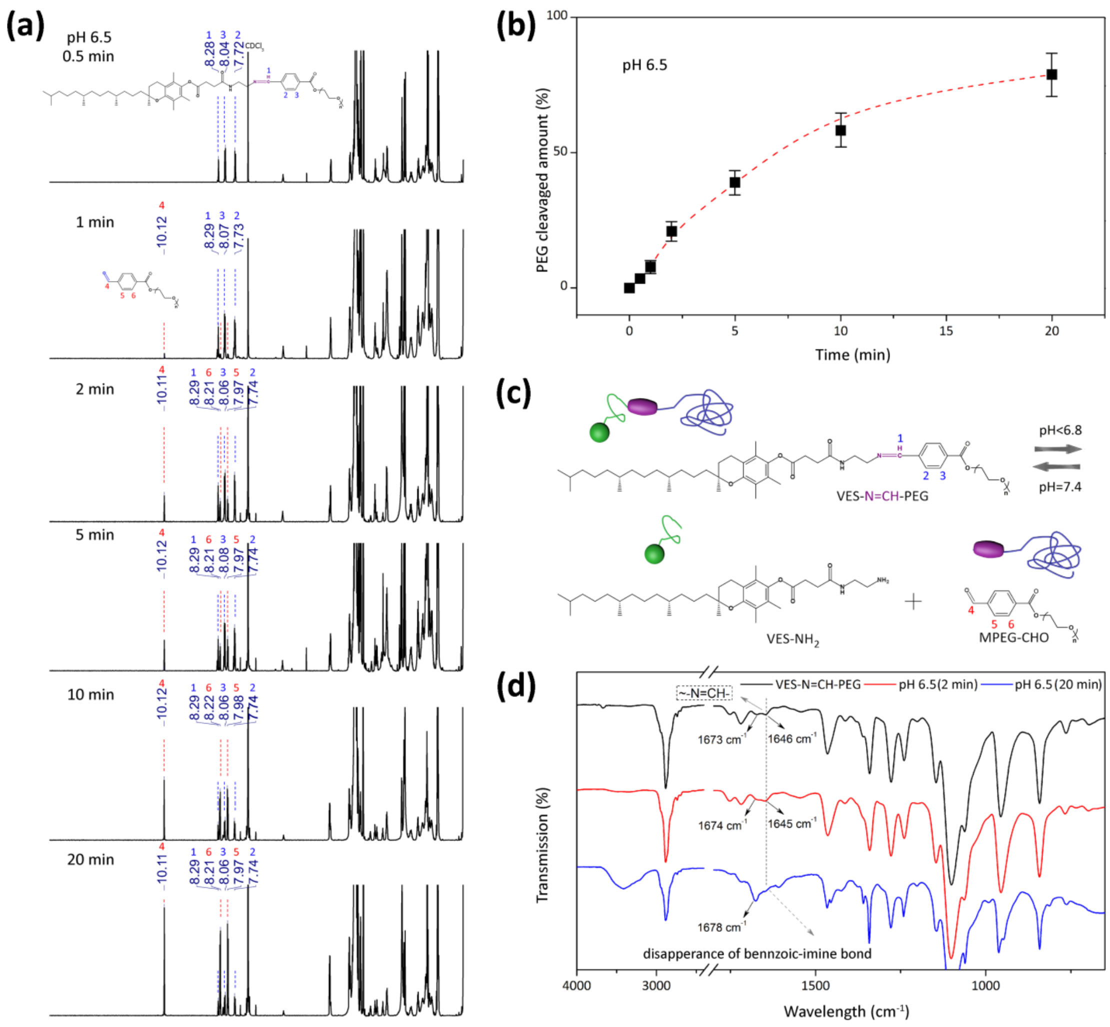

Fig. S10 a Fast variations of ^{1}H NMR spectra of VES-N=CH-PEG at pH 6.5. **b** Tumor acidity-responsive cleavage mechanism of VES-N=CH-PEG. **c** Tumor acidity-responsive cleavage behavior of VES-N=CH-PEG. **d** Fast variations of FT-IR spectra of VES-N=CH-PEG at pH 6.5

After the introduction of acidity into VES-N=CH-PEG, the aldehyde proton signal at 10.11 ppm and aromatic proton signals at 8.21 and 7.97 ppm derived from MPEG-CHO appeared and gradually strengthened during 20 min (a-c), which was due to the cleavage of VES-N=CH-PEG. The FT-IR result was well consistent with ^{1}H NMR result (d). These results demonstrated that the benzoic-imine linkage of VES-N=CH-PEG could be rapidly cleaved in response to tumor acidity.

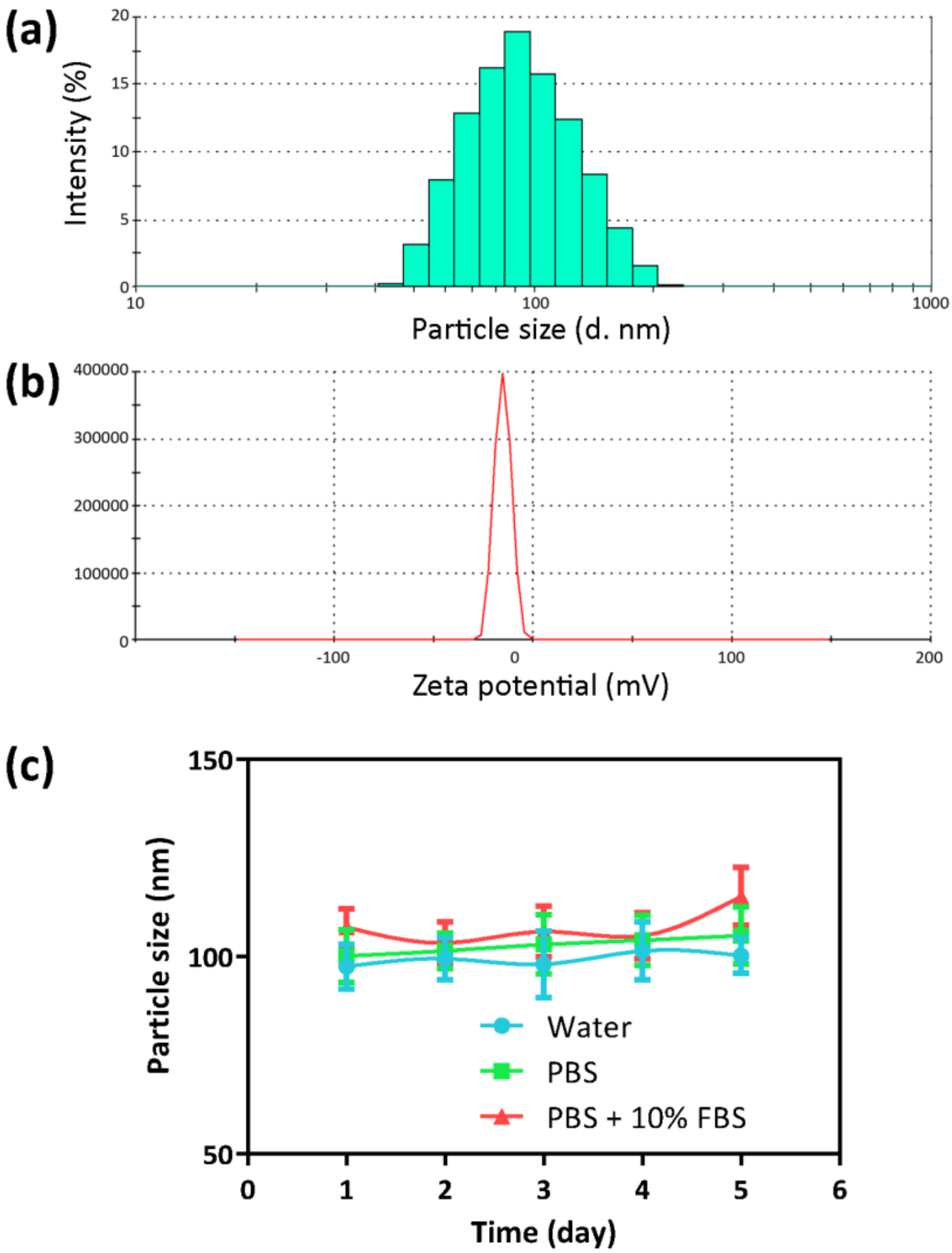

Fig. S11 a-b Hydrodynamic particle size (a) and zeta potential (b) of VSeM-N=CH-PEG. **c** Hydrodynamic particle size of VSeM-N=CH-PEG in water, PBS, and PBS with addition of 10% FBS. Error bars indicate SD (n = 3)

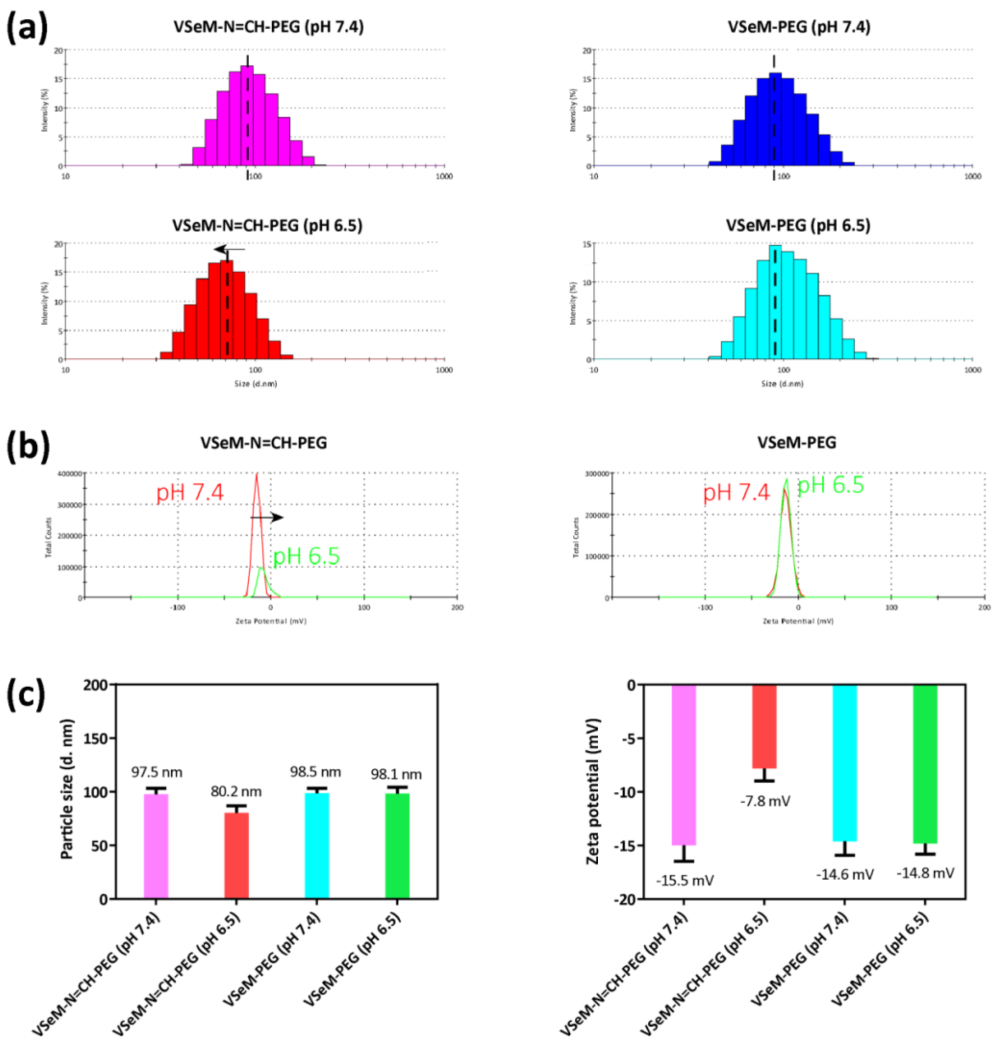

Fig. S12 a-b Hydrodynamic particle size distribution (a) and zeta potential distribution (b) of VSeM-N=CH-PEG and VSeM-PEG at both pHs of 7.4 and 6.5. **c** Variation of hydrodynamic particle size and zeta potential of VSeM-N=CH-PEG and VSeM-PEG at both pHs of 7.4 and 6.5. Error bars indicate SD (n = 3).

The VSeM-N=CH-PEG exhibited negative surface charge under physiological condition (pH 7.4). In contrast, the surface potential of VSeM-N=CH-PEG was slightly increased from ~-15.5 to ~-7.8 mV under tumor acidic condition (pH 6.5). In addition, the VES-N=CH-PEG remained the similar surface potential at pH 7.4 and 6.5. Therefore, the surface potential change of VSeM-N=CH-PEG was caused by the gradual cleavage of benzoic-imine linker and thus the detachment of PEG shell in response to the weakly acidic pH. Meanwhile, the outer PEG shell detachment could also slightly decrease the particle size of VSeM-N=CH-PEG from ~97.5 to ~80.2 nm.

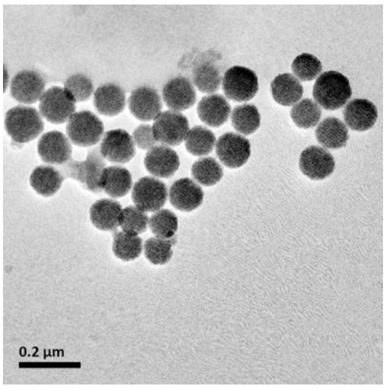

Fig. S13 TEM images of VSeM dispersed in water

The result suggested that the morphology and size of VSeM assembly and PEG-detached VSeM were basically similar.

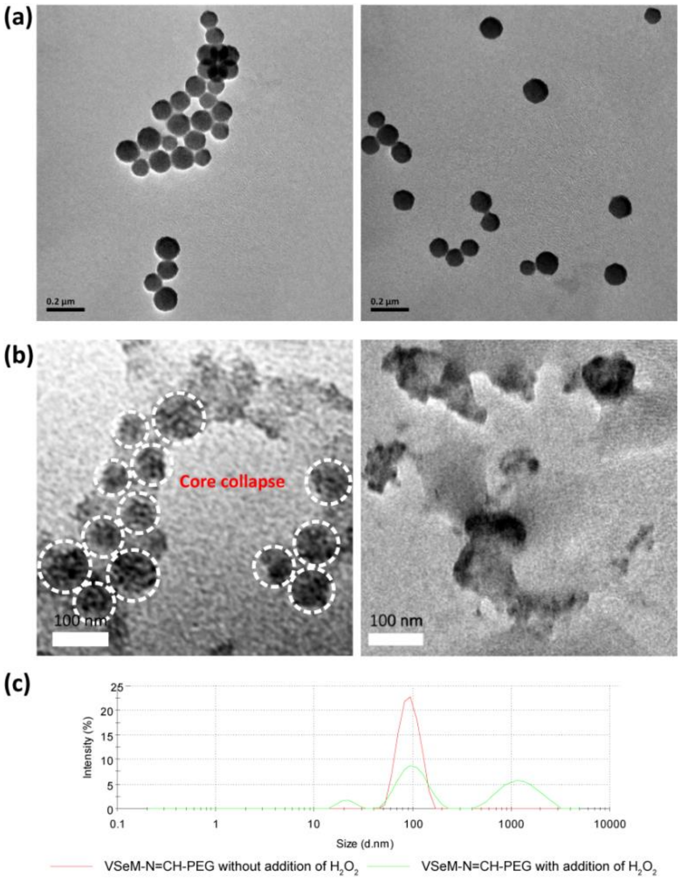

Fig. S14 a TEM images of VSeM-PEG in PBS at pH 7.4 (left) and pH 6.5 (right) with H_2O_2 for 0.5 h. **b** TEM images of VSeM-N=CH-PEG in PBS (pH 6.5) with H_2O_2 for 0.5 h (left) and 2 h (right). **c** Hydrodynamic particle size distribution of VSeM-N=CH-PEG without and with addition of H_2O_2

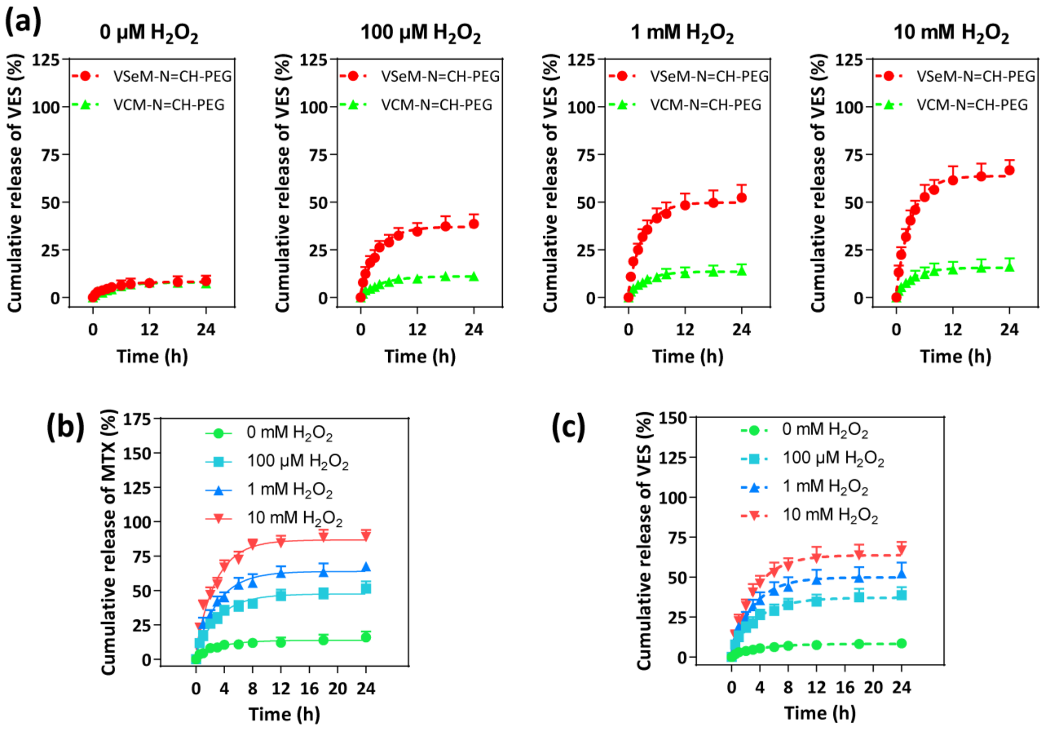

Fig. S15 a Cumulative release of VES from VSeM-N=CH-PEG and VCM-N=CH-PEG at pH 6.5 after treatment with various concentrations of H_2O_2. **b-c** Cumulative release of MTX (b) and VES (c) from VSeM-N=CH-PEG at pH 6.5 after treatment with various concentrations of H_2O_2. Error bars indicate SD (*n* = 3)

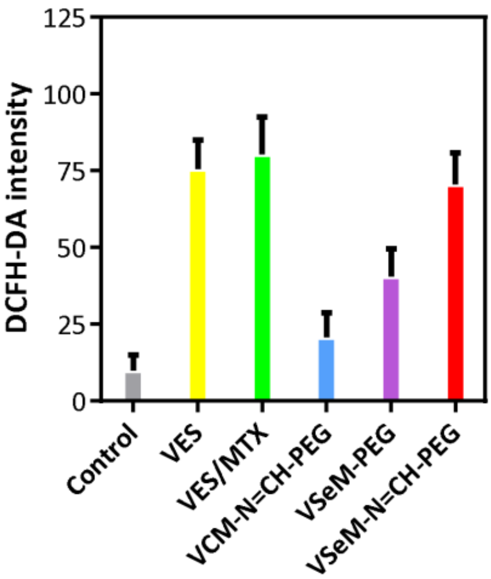

Fig. S16 Mean fluorescence intensity of ROS levels in HeLa cells treated with VES/MTX, VCM-N=CH-PEG, VSeM-PEG, and VSeM-N=CH-PEG at pH 6.5 for 6 h. ROS was stained with DCFH-DA

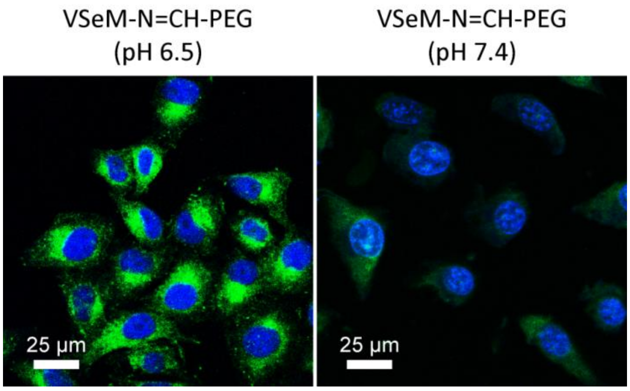

Fig. S17 CLSM images of ROS levels in HeLa cells treated with VSeM-N=CH-PEG at pH 7.4 and 6.5 for 6 h. Nuclei and ROS were individually stained with Hoechst 333258 (blue) and DCFH-DA (green)

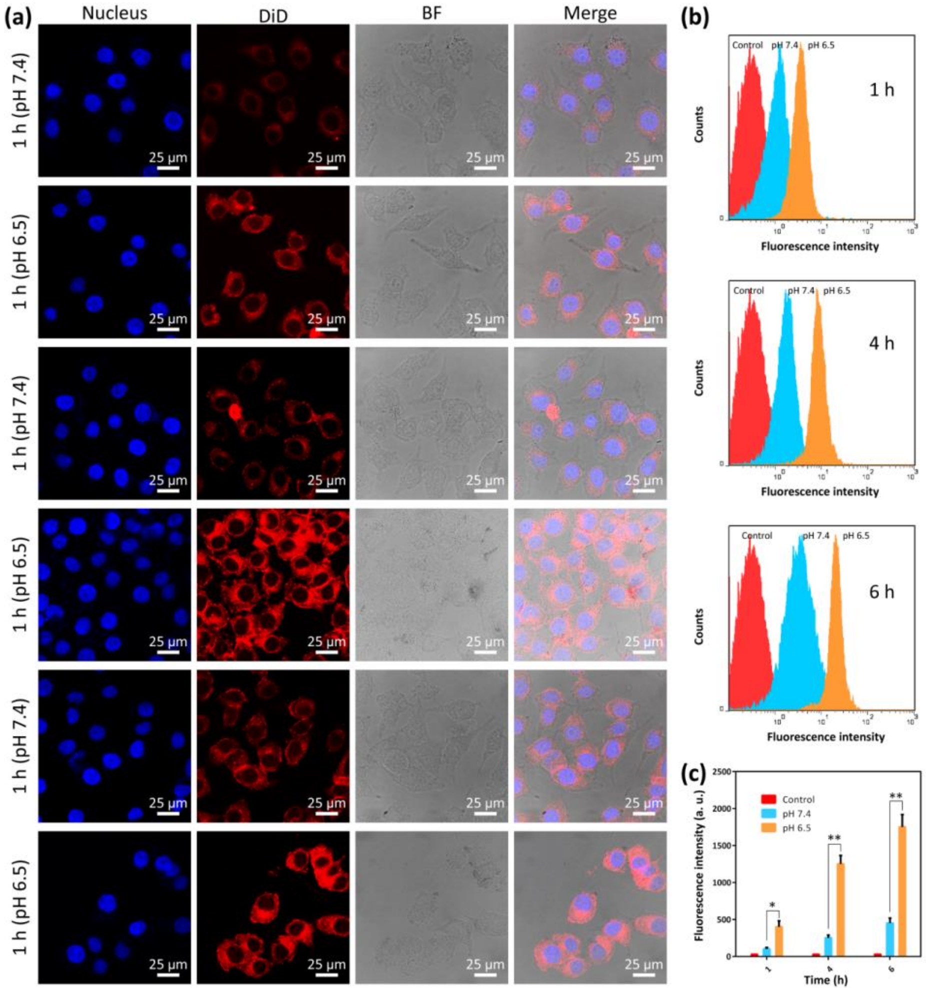

Fig. S18 CLSM images (**a**), average fluorescence intensity (**b**), and flow cytometry profiles (**c**) of HeLa cells incubated with DiD-labeled VSeM-N=CH-PEG at pH 7.4 and 6.5 for 1, 4, and 6 h. Error bars indicate SD ($n = 3$)

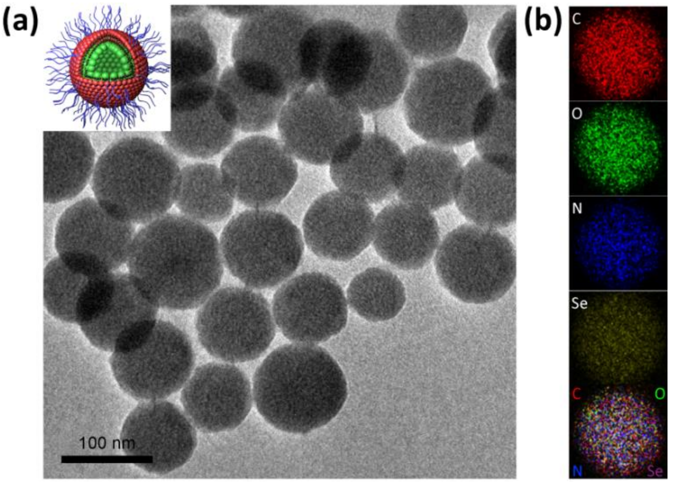

Fig. S19 TEM image and element mapping of VSeM-PEG

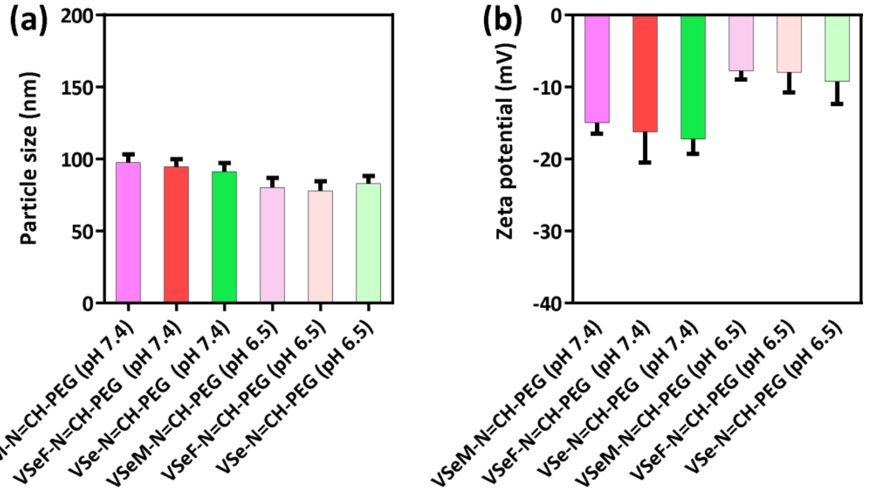

Fig. S20 Hydrodynamic particle size distribution (**a**) and zeta potential distribution (**b**) of VSeM-N=CH-PEG, VSeF-N=CH-PEG, and VSe-N=CH-PEG at both pHs of 7.4 and 6.5. Error bars indicate SD (n = 3)

Both particle size and surface charge of VSeM-N=CH-PEG, VSeF-N=CH-PEG, and VSe-N=CH-PEG nanodrugs at the same pH value (7.4 and 6.5) were similar. Thus we excluded pronounced differences in biological effects originating merely from the physicochemical characteristic of nanodrugs. So, the VSeF-N=CH-PEG and VSe-N=CH-PEG could be used as references for VSeM-N=CH-PEG.

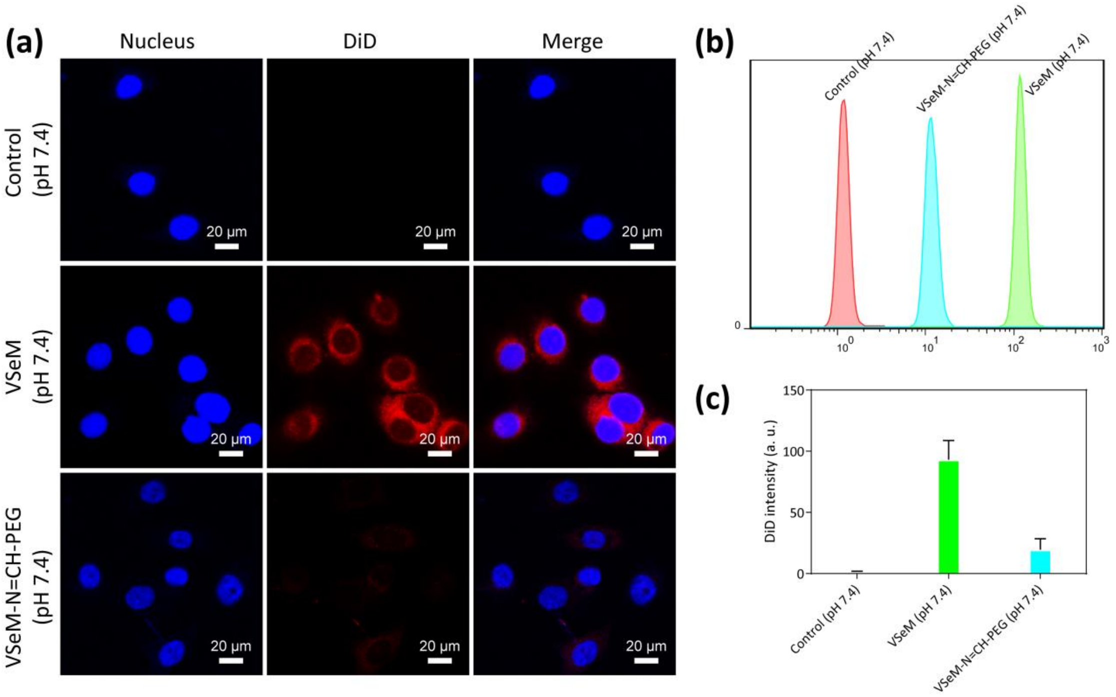

Fig. S21 CLSM images (**a**), flow cytometry profiles (**b**), and average fluorescence intensity (**c**) of RAW 264.7 cells incubated with DiD-labeled VSeM and VSeM-N=CH-PEG at pH 7.4 for 4 h. Error bars indicate SD (n = 3)

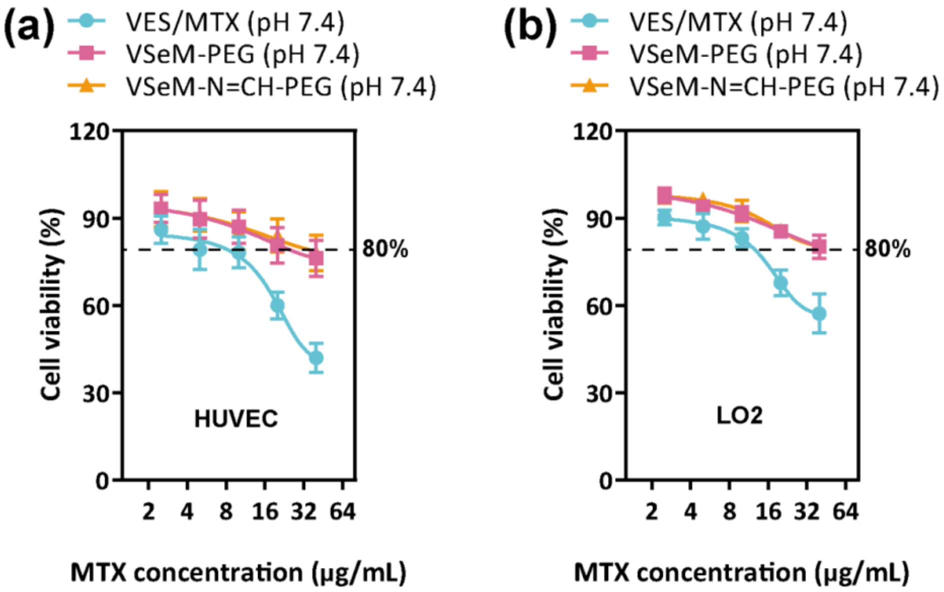

Fig. S22 *In vitro* cell viability of HUVEC and LO2 cells incubated with different concentrations of VES/MTX, VSeM-PEG, and VSeM-N=CH-PEG for 24 h. Error bars indicate SD (n = 4)

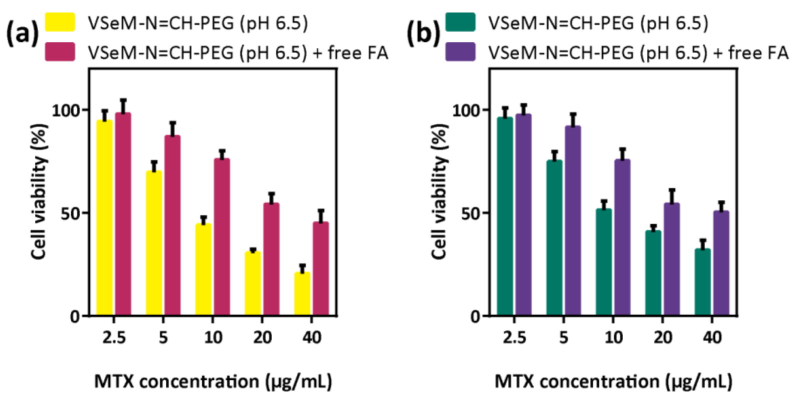

Fig. S23 *In vitro* cell viability of HeLa and MCF-7 cells incubated with different concentrations of VSeM-N=CH-PEG at pH 6.5 without and with pretreatment of free FA. Error bars indicate SD (n = 4)

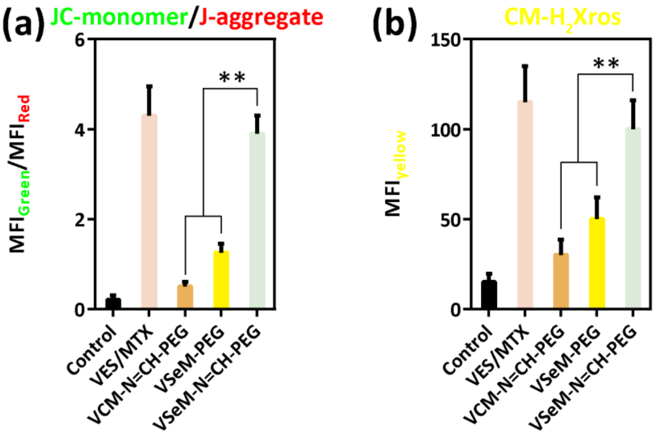

Fig. S24 Analysis of (a) mitochondrial depolarization using JC-1 and (b) cellular oxidative stress using MitoTracker Red CM-H_2Xros in HeLa cells treated with VES/MTX, VCM-N=CH-PEG, VSeM-PEG, and VSeM-N=CH-PEG at pH 6.5 for 12 h. Error bars indicate SD (n = 3)

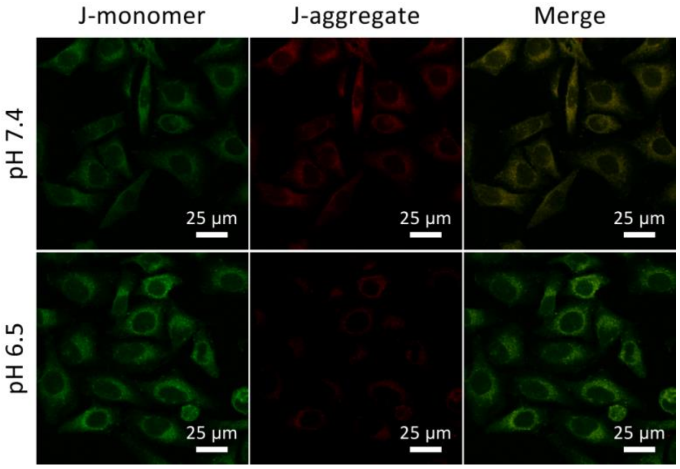

Fig. S25 CLSM images of mitochondrial depolarization in HeLa cells incubated with VSeM-N=CH-PEG at pH 7.4 and 6.5

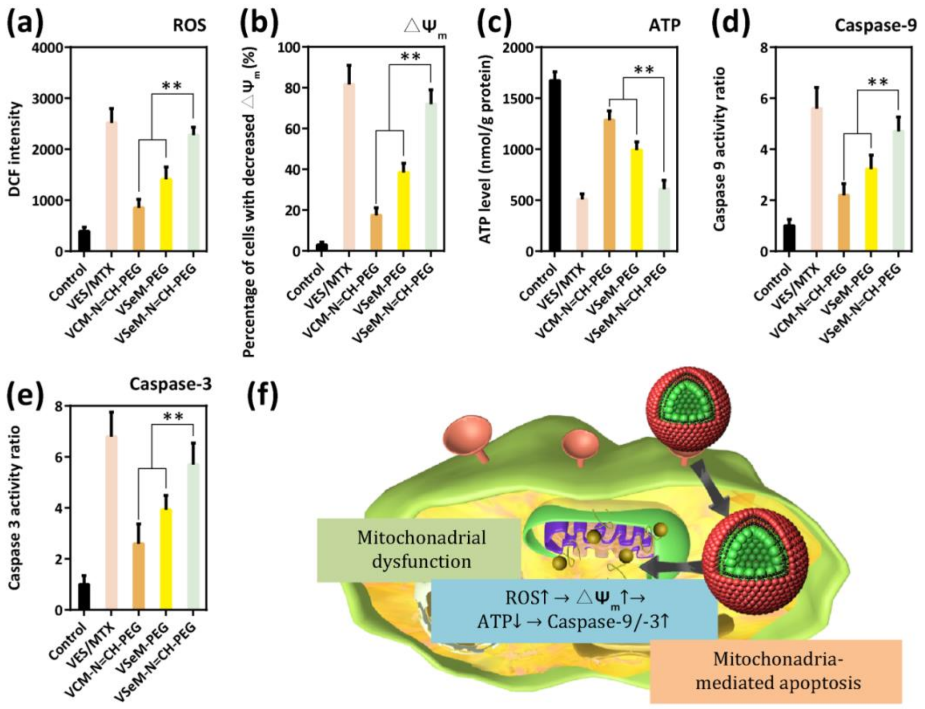

Fig. S26 a-e Analysis of intracellular ROS using DCF **(a)**, mitochondrial membrane potential (ΔΨ) using JC-1 (b), intracellular ATP levels (c), caspase-9/caspase-3 activity (d, e) in HeLa cells treated with VES/MTX, VCM-N=CH-PEG, VSeM-PEG, and VSeM-N=CH-PEG at pH 6.5 for 12 h. **f** Schematic diagram of mitochondria-mediated apoptosis in HeLa cells incubated with VSeM-N=CH-PEG upon tumor acidity

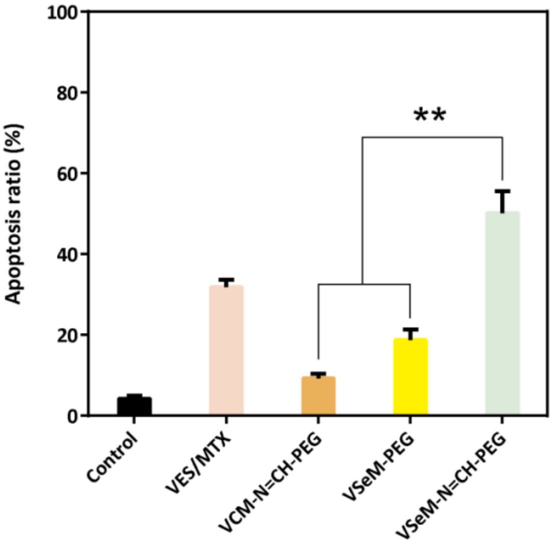

Fig. S27 Apoptosis ratio of HeLa cells treated with VES/MTX, VCM-N=CH-PEG, VSeM-PEG, and VSeM-N=CH-PEG at pH 6.5 for 12 h *via* Annexin V-FITC/PI assay. Error bars indicate SD (n = 4)

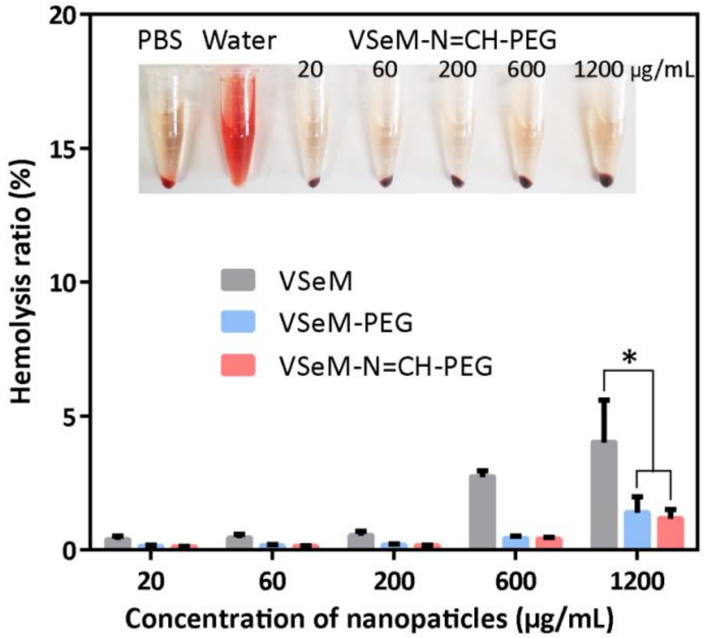

Fig. S28 Blood hemolysis test of VSeM, VSeM-PEG, and VSeM-N=CH-PEG at different concentrations. Inset: photographs of blood red cells treated with PBS, water, and VSeM-N=CH-PEG at different concentrations. Error bars indicate SD (n = 3)

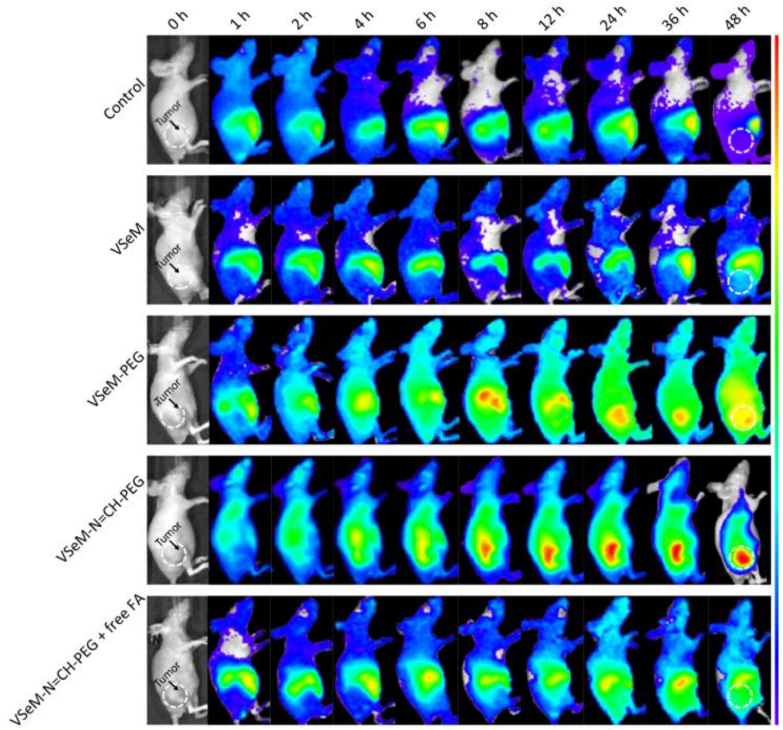

Fig. S29 Time-lapsed NIR fluorescence imaging of HeLa tumor-bearing mice

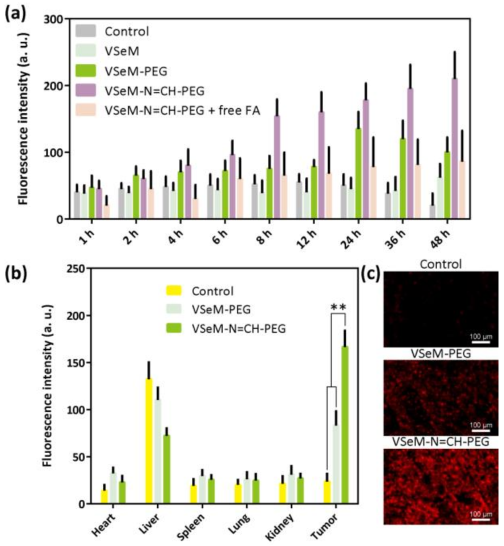

Fig. S30 a Fluorescence intensity in tumors after intravenous injection of DiR-labeled VSeM, VSeM-PEG, and VSeM-N=CH-PEG without/with pretreatment of free FA. Error bars indicate SD (n = 3). **P* < 0.05, ***P* <0.01. **b** Average fluorescence intensity of excised normal and tumor tissues of HeLa tumor-bearing mice after intravenous injection of VSeM-PEG and VSeM-N=CH-PEG. Error bars indicate SD (n = 3). **c** Frozen sections of tumor tissues after intravenous injection of VSeM-PEG and VSeM-N=CH-PEG into HeLa tumor-bearing mice for 48 h

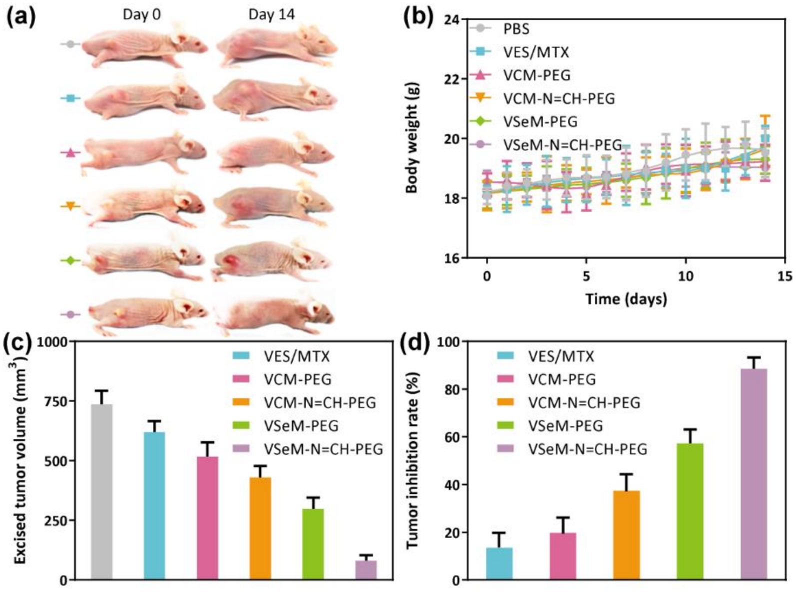

Fig. S31 a Representative photographs of HeLa tumor-bearing nude mice on the 14th day. **b** Body weight changes of HeLa tumor-bearing nude mice after different treatments. **c** Excised tumor volume of HeLa tumor-bearing mice after different treatments of VES/MTX, VCM-PEG, VCM-N=CH-PEG, VSeM-PEG, and VSeM-N=CH-PEG. **d** Tumor inhibition rate of HeLa tumor-bearing mice after different treatments of VES/MTX, VCM-PEG, VCM-N=CH-PEG, VSeM-PEG, and VSeM-N=CH-PEG. Error bars indicate SD (n = 6). ***P* <0.01, ****P* <0.005

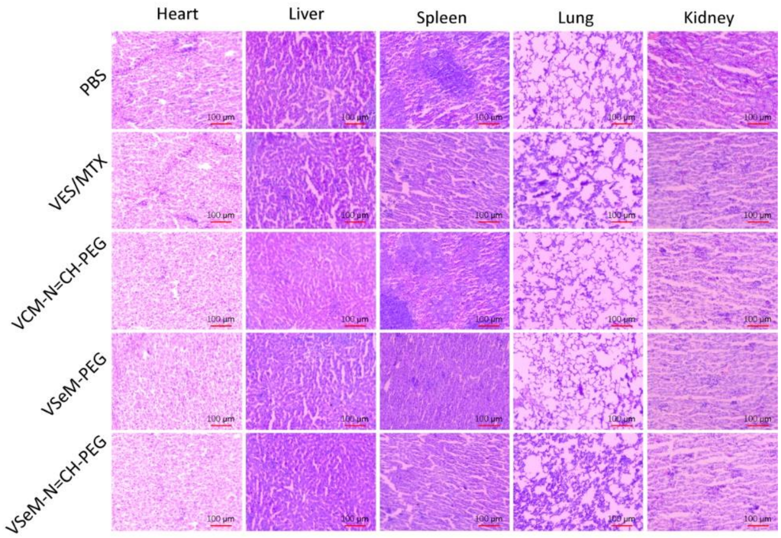

Fig. S32 Representative H&E staining histological images of major organ after different treatments of PBS, VES/MTX, VCM-N=CH-PEG, VSeM-PEG, and VSeM-N=CH-PEG

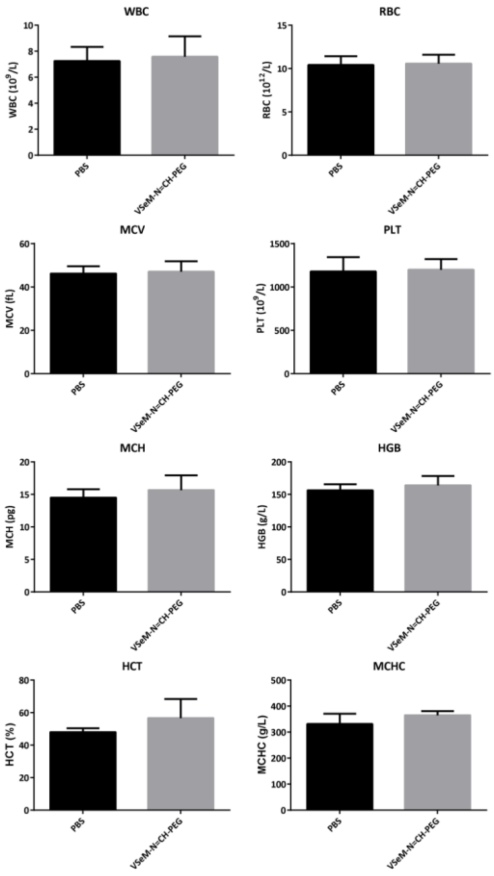

Fig. S33 Hematological indexes including white blood cells (WBC), red blood cells (RBC), mean corpuscular volume (MCV), latelets (PLT), mean corpuscular haemoglobin (MCH), haemoglobin (HGB), haematocrit (HCT), and mean corpuscular haemoglobin concentration (MCHC) of HeLa tumor-bearing mice after treatments of VSeM-N=CH-PEG on the 14th day. Error bars indicate SD (n = 6).

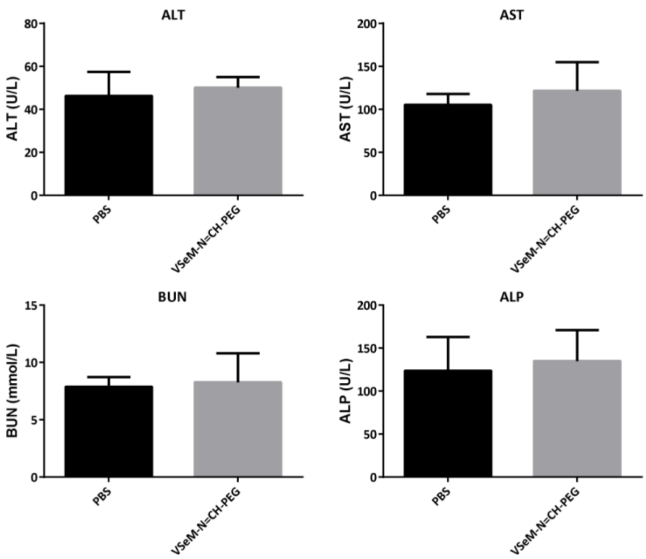

Fig. S34 Biochemical indexes including alanine aminotransferase (ALT), aspartate aminotransferase (AST), blood urea nitrogen (BUN), and alanine aminotransferase (ALP) of HeLa tumor-bearing mice after treatments of VSeM-N=CH-PEG on the 14th day. Error bars indicate SD (n = 6).
